# Sin taxes and their effect on consumption, revenue generation and health improvement: a systematic literature review in Latin America

**DOI:** 10.1093/heapol/czaa168

**Published:** 2021-04-22

**Authors:** Aurelio Miracolo, Marisa Sophiea, Mackenzie Mills, Panos Kanavos

**Affiliations:** Department of Health Policy and Medical Technology Research Group - LSE Health, The London School of Economics and Political Science, Cowdrey House, Houghton Street, London WC2A 2AE, UK; Faculty of Medicine, School of Public Health, Imperial College, London, Medical School Building, St Mary's Hospital, Norfolk Place, London W2 1PG, UK; Department of Health Policy and Medical Technology Research Group - LSE Health, The London School of Economics and Political Science, Cowdrey House, Houghton Street, London WC2A 2AE, UK; Department of Health Policy and Medical Technology Research Group - LSE Health, The London School of Economics and Political Science, Cowdrey House, Houghton Street, London WC2A 2AE, UK

**Keywords:** Sin taxes, health financing, fiscal space, Latin America, earmarked taxation, universal health coverage

## Abstract

Sin or public health taxes are excise taxes imposed on the consumption of potentially harmful goods for health [sugar-sweetened beverages (SSBs), tobacco, alcohol, among others], aiming to reduce consumption, raise additional revenue and/or improve population health. This paper assesses the extent to which sin taxes (a) can reduce consumption of potentially harmful goods, (b) raise revenue for national health systems and (c) contribute to population health in Latin America. A systematic literature review was conducted on peer-reviewed and grey literature; endpoints included: impact of raising sin taxes on consumption, ability to raise revenue for health and the possibility of population health improvements. Risk of bias for each study was assessed. The synthesis of the literature on sin tax implementation showed improvements in all three endpoints across the study countries. Following the introduction of sin taxes or by simulating their potential impact, nearly all studies explicitly reported that consumption of potentially harmful goods (mainly SSBs and tobacco) declined; revenue was found to have increased in almost all countries, suggesting that there may be additional scope for further tax increase. Simulated improvements in population health have also been shown, by demonstrating a relationship between sin tax increases and reduction in prevalence of diabetes, stroke, heart attacks and associated deaths. However, sin tax effects on health would be better quantified over the long-term. Data quality and availability challenges did place some limitations on sin tax impact assessment. Sin taxes can be effective in reducing consumption of potentially harmful goods, improve population health and generate additional revenue. Promoting further research on this topic should be a priority.

**KEY MESSAGES**

- This is the first systematic literature review assessing the effect of sin taxes on consumption, fiscal space generation and their impact on population health in Latin America.
- Reduction in harmful goods consumption (81% of studies), positive effects on revenue generation (71%) and on health outcomes (82%) are key outcomes.
- There is still room for further tax increases where sin taxes have been adopted.
- Further research is needed to improve data collection for a more comprehensive analysis of the impact of sin taxes

## Introduction

### Background

Sin taxes, or public health taxes, are defined by the World Health Organisation as excise taxes targeting goods that can be detrimental to the health of the population (WHO, 2004). These goods include tobacco products, alcohol, sugar-sweetened beverages (SSBs), which are drinks with added sugar, such as soft drinks, tea, flavoured coffee, juice and sports drinks. The harmful impact of these goods is well known and is evidenced by research (Cnossen, 2005); for instance, tobacco consumption is linked to an increased risk of developing cardiovascular disease (CVD), respiratory disease, cancer and other non-communicable and chronic diseases (U.S. Department of Health and Human Services, 2014), while elevated SSB consumption is generally associated with an increased risk of developing CVD, metabolic disease and obesity (Malik *et al.*, 2013; Arsenault *et al.*, 2017).

Published evidence has demonstrated the effect of sin taxes on consumer behaviour, health outcomes and on revenue generation for health systems (Wright *et al.*, 2017). Although differences in sin tax application and outcomes are present between low- and middle-income countries (LMICs) vs high-income countries (HICs), evidence has shown that the application of these taxes can have a significant effect on consumption patterns and the well-being of the population, while being financially sustainable (Goodchild *et al.*, 2016).

The inverse relationship between increases in sin taxes and consumption is also well established for the consumption of SSBs (Colchero *et al.*, 2017). Research related to health and behaviour connected to SSBs intake has been conducted in HICs (Claro *et al.*, 2012) reporting that consumption of SSBs instead of zero-calorie beverages can lead to excess weight and obesity. This has raised concerns over SSB consumption in LMIC settings where research is more limited.

From an economic standpoint, excise duties are a form of indirect taxation, in that they are levied on goods or services rather than on firms or personal incomes. This gives them greater capacity to shape consumer behaviour. Sin taxes can be applied in two different ways: per unit (defined as a fixed amount for each unit of a good or service sold, such as dollars per kilogram) or ad valorem (levied on spending and set as a percentage of the value added by a firm, as is the case of a value-added tax (VAT)). With the former, the tax is represented by a fixed amount per unit, while with the latter, the tax is made up of a fixed percentage per unit.

Sin taxes represent one way of raising revenue and, through that, creating fiscal space (FS). The revenue-generating capacity of sin taxes can help countries increase expenditure by creating additional FS (Heller, 2006), which, in turn, allows countries to direct financial resources to public spending without depressing other items of expenditure or by destabilizing budget equilibria.

An analytical framework of the possible policies that can be adopted for the creation of FS in the health sector has been established (Heller, 2006; PAHO, 2015); this includes, first, the promotion of conducive macroeconomic conditions; second, a reprioritization of health expenditure; third, the improvement of efficiency in existing health expenditure; fourth, increasing the efficiency of tax collection; fifth, a recourse to external aid (grants, loans); and sixth, the creation of new tax revenues through a greater tax burden (PAHO, 2015). Latin American taxation on goods such as tobacco, alcohol and sugar, which are potentially harmful for general health, is considerably lower than the average in Organization for Economic Cooperation and Development (OECD) countries (PAHO, 2015) and, as such, represents a valid policy choice for Latin American countries, since they can simultaneously generate revenue as well as influence consumer behaviour and, by implication, population health.

Latin America is considered an area with relatively high levels of consumption of products which can prove harmful to public health (tobacco, alcohol, saturated fat). Twenty per cent of people under 20 years of age are overweight or obese in the region (Cominato *et al.*, 2018), while this percentage exceeds 50% among Mexican and Peruvian adults (Kain *et al.*, 2014; Batis *et al.*, 2016; Colchero *et al.*, 2017). Furthermore, an overall high prevalence in tobacco consumption is recorded in the region: only Ecuador, Peru, Bolivia and Paraguay report a consumption of <500 cigarettes per capita per annum, while in all other Latin American countries tobacco consumption ranges between 500 and 1500 cigarettes per capita per annum (Muller, 2008). Given the significant consumption of potentially harmful goods, the associated negative impact on health in Latin America, and considering the opportunities outlined in the FS framework (PAHO, 2015), the purpose of this paper is to assess the impact of sin tax implementation in the Latin American region. A systematic literature review is conducted for this purpose. While the impact of sin taxes has been investigated at country level in some Latin American countries (Mejia *et al.*, 2008; Claro *et al.*, 2012; Curti *et al.*, 2015; Batis *et al.*, 2016) or countries outside the Latin American region (White and Ross, 2015), including HICs (Wright *et al.*, 2017), comparative evidence of this type of taxation at regional level, and, specifically, in the Latin American context, where there may be economic and cultural similarities amongst the countries in the region, is missing. While the effect of sin taxes in HICs is well established (Wright *et al.*, 2017), it is unclear if these findings translate to Latin America, where there are differences in policy priorities, policy processes and fiscal commitments. There is no study that analyses and pulls together any available evidence on the impact of sin tax introduction in Latin America, a continent dominated by middle-income countries, where public investment in health is in the majority of cases low as proportion of gross domestic product (GDP) and where increases in spending are required in order to comply with universal health coverage pledges. The paper, therefore, contributes to the discussion of whether sin taxes have any effect on tax revenue and consumption of potentially harmful products, impact health impact and, broadly speaking, contribute to healthcare financing.

## Methods

### Approach and endpoints

A systematic literature review (SLR) has been conducted to investigate the impact of sin taxes in the Latin American region. The geographical scope of the study included the South American continent, the Spanish-speaking countries of continental central America and excluded the Caribbean region. Three endpoints were considered: first, a consumption endpoint, examining whether the application of excise taxes has had any effect on the demand for goods (i.e. SSBs, unhealthy food, tobacco, alcohol); second, a revenue endpoint, which aimed to determine whether sin taxes can generate additional financial resources or FS for countries, and what priorities are defined for subsequent spending; and, third, a health impact endpoint, whose objective was to determine the role of sin taxes in changing the prevalence of diseases related to the consumption of harmful goods (i.e. CVD, diabetes, respiratory system disease, cancer and other non-communicable and chronic diseases, cardiometabolic problems, obesity or being overweight).

### Search strategy and eligibility criteria

The SLR was performed according to the Cochrane Handbook for Systematic Reviews of Interventions (Higgins *et al.*, 2019). Both peer-reviewed and grey literature sources were searched. The following databases were searched for relevant peer-reviewed literature: PubMed, ProQuest, Web of Science, Cumulative Index to Nursing and Allied Health Literature (CINAHL) and EconLit. The goal of the grey literature search was to identify publications from intergovernmental organizations that were relevant to and/or offered information and insights on our endpoints. Relevant grey literature was identified through the OECD, the International Monetary Fund (IMF), the World Bank, the World Economic Forum (WEF), the World Health Organisation (WHO) and the Pan-American Health Organisation (PAHO) databases. The search and screening process commenced in May 2018 and was completed in February 2019. Search terms included:

‘Sin Tax*’ OR ‘Sugar Tax*’ OR ‘Tobacco Tax*’ OR ‘Alcohol Tax*’ OR ‘Salt Tax*’ OR ‘Sodium Tax*’ OR ‘Excise Tax*’ OR ‘Food Tax*’ OR ‘Earmark* Tax*’ OR ‘Cigarette Tax*’ OR ‘Beer Tax*’ OR ‘Wine Tax*’ OR ‘Beverage Tax*’ OR ‘Calorie Tax*’ OR ‘Processed Food Tax*’

*AND*

*‘Latin America’ OR ‘South America’ OR ‘Central America’ OR ‘Argentina’ OR ‘Belize’ OR ‘Bolivia’ OR ‘Brazil’ OR ‘Brasil’ OR ‘Chile’ OR ‘Colombia’ OR ‘Costa Rica’ OR ‘Ecuador’ OR ‘El Salvador’ OR ‘French Guiana’ OR ‘Guatemala’ OR ‘Guyana’ OR ‘Honduras’ OR ‘M***_*é*_***xico’ OR ‘Mexico’ OR ‘Nicaragua’ OR ‘Panama’ OR ‘Paraguay’ OR ‘Peru’ OR ‘Suriname’ OR ‘Uruguay’ OR ‘Venezuela’*

This search strategy included all terms for sin taxes used in Latin American countries, the range of different goods on which taxes are usually applied, and all countries within the Latin American region. The intervention related to the application of sin taxes on harmful goods such as tobacco or high energy density foods, with different outcomes, all subsequently classified under one of the three defined endpoints. Relevant publications in English and Spanish were included. The exclusion steps considered were (1) exclusion of duplicates (as soon as they were identified through the screening process), (2) exclusion of unrelated titles, (3) exclusion of unrelated abstracts and (4) exclusion through full-text analysis.

Study exclusion criteria were non-Latin American countries, previous systematic literature reviews or previous meta-analyses, books or chapters of books, dissertations and theses, presentation abstracts, studies not related to any of the considered endpoints and studies lacking any assessment of relevant taxes. The study period ranged from 1 January 2000 to 31 December 2018. Table 1 summarizes the Population, Intervention, Comparator, Outcome, Study Design, Time frame (PICOST) characteristics.

**Table 1:** PICOST table

Component	Criteria
Population	Countries in Latin America: Argentina, Belize, Bolivia, Brazil, Chile, Colombia, Costa Rica, Ecuador, El Salvador, French Guiana, Guatemala, Guyana, Honduras, Mexico, Nicaragua, Panama, Paraguay, Peru, Suriname, Uruguay and Venezuela.
Intervention	Tax implementation and simulation on ‘harmful’ goods (alcohol, sugar, salt, junk food (i.e. calorie-dense foods) and/or tobacco products).
Comparison	No direct comparator for this study. However, studies may identify the differences in health outcomes, consumption and revenue generation before and after the sin tax was introduced on tobacco, salt, sugar and/or alcohol, which would provide further information on the effect of sin taxes on the outcomes of interest.
Outcome	To investigate how the sin taxes on sugar, salt, tobacco, alcohol and calorie-dense foods can affect health outcomes, consumption and revenue generation across Latin American countries.
Study design	Peer-reviewed and grey literature will be eligible for inclusion in the review, so long as they fit into the research criteria and outcomes of interest.
Time	From inception of PubMed, ProQuest, Web of Science, CINAHL and EconLit, Grey literature (OECD, WHO, World Bank, IMF, WEF and PAHO) to the last week of December 2018.
Outcomes not included	Any studies in non-Latin American countries and/or other systematic reviews or meta-analyses, books or chapters of books, dissertations and theses, presentation abstracts.
Selection criteria for full-text screening	Adherence to the intervention and the outcome of interest.

### Data extraction

In accordance to Cochrane guidance (Higgins *et al.*, 2019), a template to organize the identified information has been implemented. An initial template, drawn up in Excel, included all the studies that resulted after a first screening of duplicates and titles. This template included information on the main characteristics of each study (title, author(s) and country or location), data on the purpose of the study and the tax of interest, number of participants, participant characteristics, the investigated endpoint(s), findings of the evaluation and a brief statement on the conclusion of the study. This step has been key in assisting a further screening process through the abstract analysis and, in the final stage, through the evaluation of full-text features.

### Risk of bias assessment criteria

A risk of bias assessment was performed during the research for full-text evaluation, according to the ROBINS-I tool (Sterne *et al.*, 2016) developed by Cochrane and the BMJ, with the goal of defining the quality of the studies. The domains included for the risk of bias assessment related to confounding, selection of participants into the study, deviations from intended interventions, missing data, measurement of outcomes and selection of the reported results.

### Data synthesis

Findings are grouped under the three endpoints, (1) effect on consumption, (2) effect on revenue generation and (3) health impact. In each of the three endpoints, there was a further division, where possible, relating to the type of good (e.g. SSBs, tobacco, alcohol). The study's PROSPERO identification number is CRD42018096210.

## Results

### Study characteristics

**Figure 1:**
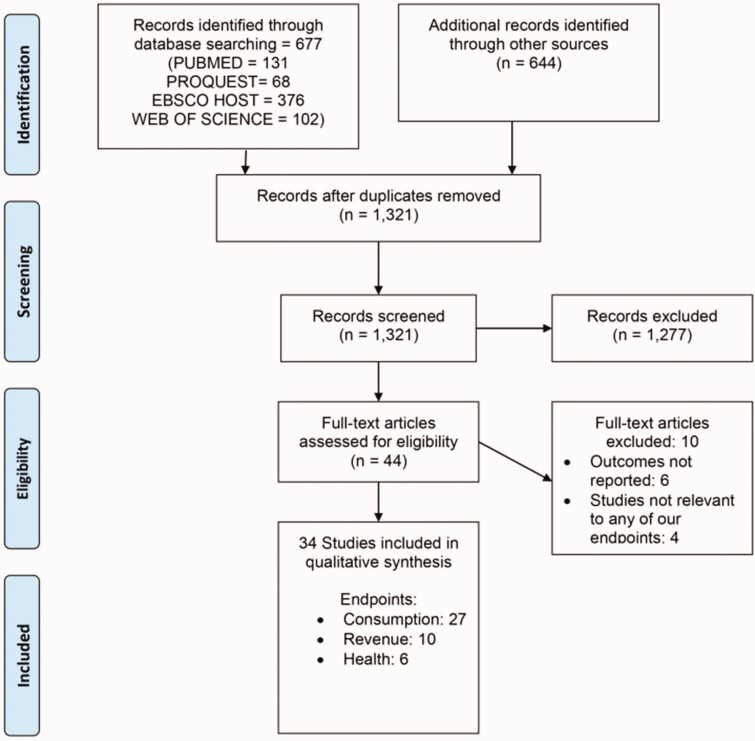
PRISMA flowchart.

The PRISMA flowchart (Figure 1) shows the number of studies included in our review and how they are arrived at. In the initial stage of the systematic review, 1321 studies were found across all databases. Following the screening process and by applying the exclusion criteria, 34 studies were included in the review.

Of the 34 included studies, 27 addressed the consumption endpoint, 6 the health endpoint and 10 the revenue generation endpoint; 9 studies addressed multiple endpoints. There were no randomized control trials (RCTs) amongst the included studies.

With regards to the intervention, 13 studies focused on SSBs and high energy density foods. This included the excise tax on SSBs (1 peso/L) and the 8% sales tax on foods implemented in Mexico in January 2014 and the SSBs excise tax in Brazil. Twenty-three studies were related to the taxation of tobacco products. Countries involved in the analysis included Mexico, Argentina, Brazil, Uruguay, Ecuador, Peru, Colombia and Panama. Two studies analysed the intervention on a continental and multi-country level (Garcés *et al.*, 2014; Goodchild *et al.*, 2017). Studies on tobacco focused mainly on the change in demand for tobacco, the impact on price caused by the tax implementation and the main features related to the demographic and epidemiological context in which these policies are operating. Alcohol was assessed in just one study, together with the analysis of tobacco demand in Ecuador (Chavéz, 2016).

The SLR included mostly observational studies and to a lesser extent narrative reviews. Most of the included literature focused on studies analysing consumption, and the main goods of interest were, first, tobacco, its demand, and the role of illicit trade and, second, SSBs and their impact on all three endpoints. Studies displayed significant variety in the population included, data sources, and evaluation methods for the specific tax of interest, as well as the evaluation of the specific tax of interest. Country differences in taxation systems, sin tax structure and levels of stakeholder involvement have added complexity to our analysis. The dominance of observational studies and the absence of other study designs (e.g. RCTs) is the result of the type of argument addressed and the requirement of wide population cohorts, which represent the national trend and must not be criticized as a source of low-quality evidence (Pindyck *et al.*, 2018). Table 2 outlines the characteristics of included studies (endpoint, publication outlet, national setting, population, data sources indicator of interest).

**Table 2:** Study key characteristics

Author(s)	Endpoint	Year	Journal	Setting	Number of participants	Data source	Participant age	Tax/indicator of interest
Studies adopting observational data								
Álvarez-Sánchez *et al.* (2018)	Consumption	2018	*PLoS One*	Mexico	6,650 individuals	National Health and Nutrition Survey 2016	Ages between 20-59 years	SSBs
Batis *et al.* (2016)	Consumption	2016	*PLoS Medicine*	Mexico	6,248 households	Mexico Consumer Panel Services (CPS)	General population	SSBs
Caro *et al.* (2018)	Consumption	2018	*PLoS Medicine*	Chile	2,000 households	Kantar Worldpanel Chile	General population	SSBs
Chavéz (2016)	Consumption	2016	*Pan American Journal of Public Health*	Ecuador	39,617 households	National Survey of Urban and Rural Household Income and Expenditures (ENIGHUR)	Population of ≥5 years of age	Demand for tobacco
Claro *et al.* (2012)	Consumption	2012	*American Journal of Public Health*	Brazil	48,470 households	Household Budget Survey (HBS)	General population	SSBs
Colchero *et al.* (2016a)	Consumption	2016	*PLoS One*	Mexico	Sales data from the Monthly Surveys of the Manufacturing Industry (EMIM) are adopted. Data on number of participants are not available	Monthly Surveys of the Manufacturing Industry (EMIM)	Sales data from the Monthly Surveys of the Manufacturing Industry (EMIM) are adopted. Data on participants age are not available	SSBs
Colchero *et al.* (2016b)	Consumption	2016	*British Medical Journal*	Mexico	6,253 households	Nielsen Mexico’s Consumer Panel Services	General population	SSBs
Colchero *et al.* (2017)	Consumption	2017	*Health Affairs*	Mexico	6,645 households	Nielsen Mexico’s Consumer Panel Services	General population	SSBs
Curti *et al.* (2015)	Consumption	2005	*Tobacco Control*	Uruguay	12,591 individuals, collected in four waves of a survey	International Tobacco Control (ITC) Evaluation Project (ITC Uruguay) survey	Adults of ≥18 years of age	Tobacco excise tax
Ferrante *et al.* (2007)	Consumption/Health	2007	*Pan American Journal of Public Health*	Argentina	22,910 individuals	Department of Statistics of the Ministry of Health of Argentina	Population of ≥15 years of age	Tobacco excise tax
Garcés *et al.* (2014)	Revenue	2014	*Nicotine & Tobacco Research*	Central America	Discussion on tobacco taxes in Central America. Number of participants is not specified	Not applicable	Discussion on tobacco taxes in Central America. Participants’ age is not specified	Tobacco
Gonzalez-Rozada and Ramos-Carbajales (2016)	Consumption	2016	*Pan American Journal of Public Health*	Peru	35,000 households	Two datasets from the National Institute of Statistics and Informatics of Peru	General population	Tobacco
Goodchild *et al.* (2017)	Consumption/revenue	2017	*Pan American Journal of Public Health*	Latin America and Caribbean	Data on number of participants not specified. Data on taxes and prices for a 20-cigarette pack were derived from the WHO report on the global tobacco epidemic. Share of licit (i.e. tax paid) cigarette retail sales in each country was collected from GlobalData and Euromonitor	WHO Bulletin 2016, WHO report on the global tobacco epidemic, Global Data and Euromonitor International	Data on participants’ age not specified.	Tobacco excise taxes
Guerrero-Lopez *et al.* (2013)	Consumption	2013	*Salud Publica de Mexico*	Mexico	66,684 individuals from ENSA 2000; 70,297 individuals from ENSANUT 2006; and 67,786 individuals from ENSANUT 2012	Encuesta Nacional de Salud (ENSA) 2000; Encuesta Nacionales de Salud y Nutriciòn (ENSANUT) 2006 and 2012	Population of ≥10 years of age	Tobacco
Hernández *et al.* (2019)	Consumption	2019	*Preventive Medicine*	Mexico	154,777 individuals, collected in five waves of ENIGH survey (2008, 2010, 2012, 2014 and 2016)	National Household Income and Expenditure Surveys (ENIGH)	General population	8% tax on energy-dense, nutrient-poor foods
Iglesias (2016)	Consumption/revenue	2016	*Revista Sudamericana de Salud Publica*	Brazil	Not applicable	Not applicable	Not applicable	2011 tobacco tax
Iglesias *et al.* (2017)	Consumption/revenue	2016	*Tobacco Control*	Brazil	37,317 individuals	GATS (Global Adult Tobacco Survey) 2008 and 2013 survey	Adults of ≥18 years of age	2012 cigarette excise tax
Jan *et al.* (2014)	Health	2014	*PLoS One*	Panama	2,191 individuals	National Institute of Statistics and Census of Panama	Population of ≥30 years of age	TTI
Kostova *et al.* (2014)	Revenue	2014	*Tobacco Control*	Brazil, Mexico, Uruguay	45,838 individuals	GATS (Global Adult Tobacco Survey)	Population of ≥15 years of age	Tobacco
Maldonado *et al.* (2016)	Consumption	2016	*Pan American Journal of Public Health*	Colombia	Estimates on country population	National Directorate of Taxes and Customs of Colombia	Population of ≥15 years of age	Tobacco
Martinez *et al.* (2015)	Consumption	2013	*British Medical Journal*	Argentina	Number of participants not specified. Cigarette consumption data derived from total sales of cigarettes to the public reported by the Ministry of Economics and Production. Population data were collected from the INDEC	Ministry of Economics and Production of Argentina; National Institute of Statistics and Census (INDEC)	Adults of ≥14 years of age	Tobacco tax
Nakamura *et al.* (2018)	Consumption	2018	*PLoS Medicine*	Chile	2,836 households	Kantar Worldpanel	General population	SSBs
Ng *et al.* (2019)	Consumption	2018	*Public Health Nutrition*	Mexico	6,089 households	Nielsen’s Mexico Consumer Panel Services	General population	SSBs
Ortega-Avila *et al.* (2018)	Consumption	2018	*Public Health Nutrition*	Mexico	29 individuals	Semi-structured interviews	Adolescents (from 15–19 years old)	SSBs
Rodriguez-Iglesias *et al.* (2016)	Consumption	2016	*Argentine Journal of Cardiology*	Argentina	Number of participants not specified. Intensity and prevalence of tobacco use were derived from the National Risk Factor Survey	National Risk Factor Survey (2005, 2009 and 2013)	Participants’ age not specified.	Tobacco tax
Saenz-de-Miera *et al.* (2010)	Consumption	2010	*Tobacco Control*	Mexico	1,079 individuals	International Tobacco Control Policy Evaluation Survey (ITC-Mexico)	Adults of ≥18 years of age	Tobacco
Szklo *et al.* (2018)	Consumption	2018	*American Journal of Public Health*	Brazil	Gathers information from different surveys	GATS (Global Adult Tobacco Survey) Brazil; VIGITEL; Secretariat of Federal Revenues of Brasil	General population	Illicit tobacco consumption
Taillie (2017)	Consumption	2017	*Preventive Medicine*	Mexico	6,089 households collected in three waves	Nielsen Company’s Mexico Consumer Panel	General population	SSBs

Studies using simulation techniques								

Bardach *et al.* (2016)	Health/revenue	2016	*Revista Peruana de Medicina Experimental y Salud Pùblica*	Peru	30,000,000 observations (individuals as part of a Monte Carlo simulation)	Databases (Medline, Embase, Central, SocINDEX, Econlit, LILACCS, NBER, CRD)	Adults of ≥35 years of age	Tobacco
James *et al.* (2019)	Health/revenue	2019	*Tobacco Control*	Colombia	Number of participants not specified. Median cigarette price obtained from the Department of National Statistics; cigarette sales were derived from ENCV and EAM	Administrative Department of National Statistics of Colombia, Encuesta Nacional de Calidad de Vida (ENCV) and Encuesta Anual Manufacturera (EAM)	Participants age’ not specified. Median cigarette price obtained from the Department of National Statistics; cigarette sales were derived from ENCV and EAM	Tobacco tax
Jiménez-Ruiz *et al.* (2008)	Consumption/revenue	2008	*Tobacco Control*	Mexico	109,089 households, collected in seven waves of ENIGH survey (1994, 1996, 1998, 2000, 2002, 2004, 2005)	National Household Income and Expenditure Survey (ENIGH)	General population	VAT
Reynales-Shigematsu *et al.* (2015)	Consumption/health	2015	*Pan American Journal of Public Health*	Mexico	11,072 individuals from ENA 2002 survey; 16,249 individuals from ENA 2011 survey. Population with 65 years old or older obtained from ENSANut 2000, however, number of individuals not specified; 13,627 individuals from GATS survey; ∼3,000 individuals from six waves of ITC Mexico survey	National Survey of Addictions (ENA); National Enquiry of Health and Nutrition (ENSANut); Global Adult Tobacco Survey (GATS); International Tobacco Control Policy Evaluation Survey (ITC Mexico)	Adults from 12 to 65 years of age (ENA); adults with more than 65 of age (ENSANut); Adult smokers from 18 years of age (ITC-Mexico); Smokers from 16 years of age (GATS)	Tobacco tax
Rodríguez-Iglesias *et al.* (2017)	Consumption/revenue	2017	*Salud Publica de Mexico*	Argentina	Monthly data on cigarette sales and average weighted prices were collected from the Ministry of Agriculture. Average nominal income of the private sector was derived from the MECON	Ministry of Agriculture of Argentina, Ministry of Economics (MECON) of Argentina	Not applicable	Tobacco
Sánchez-Romero *et al.* (2016)	Health/revenue	2016	*PLoS Medicine*	Mexico	2,338 individuals	National Enquiry of Health and Nutrition 2012 (ENSANUT)	Adults from 35 to 94 years of age	SSBs

TTI, Tobacco tax increase; SSB, sugar-sweetened beverage; SPST, special production and services tax; VAT, value-added tax.

### Effect on consumption

#### SSBs and unhealthy foods

The effect of sin taxes on consumption of SSBs was addressed by 12 studies. Nine of these were related to the implementation of SSBs taxes in Mexico, two focused on taxation of high-sugar content beverages in Chile and one investigated the potential relationship between SSB prices and levels of consumption in Brazil.

The literature focused on Mexico due to the high levels of SSB consumption. Before tax implementation, Mexico had the highest worldwide soft drinks consumption (163 litres per capita) in 2011 (Colchero *et al.*, 2016b). In January 2014, Mexican government introduced a tax of 1 Mexican peso per litre on all sugary non-alcoholic beverages, i.e. sodas, flavoured waters, sweetened dairies, teas and energy drinks with added sugars, but excluded drinks consisting of 100% juice and beverages with artificial sweeteners (Claro *et al.*, 2012). This caused an 11% price increase in carbonated SSBs and circa a 10% price increase in non-carbonated SSBs, compared with prices in 2013 (Colchero *et al.*, 2016a). At the same time, Mexico introduced an 8% ad valorem tax on non-essential highly energy-dense foods (with at least 275 calories per 100 g) (Colchero *et al.*, 2016b).

Six studies analysed the changes in consumption caused by the implementation of the SSB tax (1 peso/l) in Mexico. The common aim of these studies was to understand how consumer behaviour would change following the tax introduction. This was achieved by investigating different data sources, notably, Nielsen's Mexico Consumer Panel services (henceforth Nielsen Panel), that collects data on households' monthly purchases and covers 63% of the Mexican population, and the Mexican National Health and Nutrition Survey based on questionnaire responses and manufacturing sector data, particularly the ‘Economic Behaviour of the Industries in the Country’ (EMIM) database. All six studies highlighted that the introduction of the specific SSB tax increased the price of SSB products approximately by 10% in 2014 compared with 2013. Results from one study (Colchero *et al.*, 2017) showed a decrease in SSB purchases of 5.5% in 2014 and 9.7% in 2015 (average reduction of −7.6% in 2014–15) compared with the 2012–13 period. Another study (Colchero *et al.*, 2016b) based on the same source found a change in SSB purchases of −6% in 2014 compared with 2012–13. The reduction was higher in low socioeconomic status (SES) groups, relative to medium and high SES groups (−9.1% vs −5.5% vs −5.6%, respectively). Another study (Ng *et al.*, 2019), based on the Nielsen Panel, divided the study population in four groups encompassing all possible consumers of taxed and untaxed beverages: (1) those who had higher (H) purchases of taxed (T) beverages and lower (L) purchases of untaxed (U) beverages (HTLU—and whose consumption choices were considered unhealthier), (2) those who had higher (H) purchases of taxed (T) and higher (H) purchases of untaxed (U) beverages (HTHU—whose consumption choices were also considered unhealthier), (3) those who had lower (L) purchases of taxed (T) and lower (L) purchases of untaxed (U) beverages (LTLU—whose consumption choices were considered healthier) and (4) those who had lower (L) purchases of taxed (T) beverages and higher (H) purchases of untaxed (U) beverages (LTHU—whose consumption choices were also considered healthier). The study compared the pre-tax behaviour of these groups with their consumption levels after the SSB tax implementation. Among others, results showed that, following the SSB tax implementation, the HTLU-unhealthier and HTHU groups (both considered to be ‘unhealthy’ in their consumption choices), reduced their consumption of taxed beverages both in absolute and relative terms and, at the same time, increased their consumption of untaxed beverages. It has been shown that the greatest effect of this consumption shift from taxed to untaxed beverages was observed in the lowest socioeconomic group. A further study (Colchero *et al.*, 2016a) using an alternative data source, notably, manufacturing industry data (EMIM) analysed the changes in SSB and plain water sales in 2014 and 2015 (using the pre-tax period, 2007–13, as a counterfactual). Results suggested a decrease in SSB per capita sales of 7.3% and an increase of 5.2% in plain water per capita sales in the 2014–15 period compared with the counterfactual, reporting an association of the tax implementation with the changes in per capita sales. Overall, results of the studies assessing SSB tax implementation in Mexico reported a decrease in the consumption of taxed SSBs, and that the tax mildly shifted purchases towards untaxed beverages or other products. Some studies (Colchero *et al.*, 2016b, 2017; Wright *et al.*, 2017) pointed out that effects of tax implementation may be more substantial in the long-term rather than the short-term. This would be because human habit formation is gradual and changing behaviour in light of increased taxation may take time to shape (Colchero *et al.*, 2017; Wright *et al.*, 2017). Additionally, following tax implementation consumers may switch to cheaper untaxed beverages and this pattern could be better seen over the longer term (Colchero *et al.*, 2016b). The results (measures, intervention and counterfactual) included in the above studies were adjusted for different indicators, mainly seasonality of beverage consumption and socioeconomic factors. Without such adjustments, the results would have been biased by temporary factors.

Ortega-Avila *et al.* (2018) examined how the implementation of the tax was perceived by a cohort of adolescents. A qualitative study explored the awareness and perception on the introduction of the SSB tax within a cohort of Mexican adolescents, reported most of them were unaware of this policy and that they perceived the 1 peso/l increase as not high enough to shift their preferences and SSB consumption patterns. For those interviewed, alternatives to costly SSB products would mainly be homemade drinks. The study underlined that the impact of the tax could be misperceived by some segments of the population and that this would represent a limitation in changing citizens’ attitudes towards these products. Another study (Álvarez-Sánchez *et al.*, 2016) focused on the awareness of Mexicans on the SSB tax introduction. Based on questionnaire survey data of >6,000 adults, the study found that people’s awareness and decrease in consumption were directly proportional, i.e. people who were aware of the tax introduction were more inclined to decrease their SSB intake.

Three studies (Batis *et al.*, 2016; Taillie *et al.*, 2017;Hernández *et al.*, 2019) focused on the 8% ad valorem tax on non-essential and energy foods in Mexico. One study (Batis *et al.*, 2016) analysed the difference in the volume purchase of taxed and untaxed packaged food between observed data in 2014 and their respective counterfactual (2012–13). The study showed that, in 2014, the consumption on purchased food was 467 g/per capita/year, compared with the 492 g consumed food predicted by the counterfactual, with the mean volume of taxed food purchases decreasing by 5.1%. At the same time, non-significant variation was found between observed and counterfactual volumes of untaxed food purchases. A difference in consumption between SES groups was detected as well. For the low SES, there was a decrease of 10.2%, while for medium SES the decrease stood at 5.8%. Interestingly, no change in consumption was found in the high SES. However, the study pointed out that it was difficult to infer a causality between the tax implementation and the consumption changes due to database limitations in terms population representativeness (data mainly concentrated in urban areas), and the 2 years’ counterfactual could be considered limited in evidencing changes in consumption patterns. Results from the second study (Hernández *et al.*, 2019) were in the same direction, recording a decrease of 5.3% on taxed food purchases in 2014–16 compared with 2008–12. At the same time, untaxed food consumption increased by 2.8% during the same period.

The last study focused on the 8% ad valorem tax in Mexico (Taillie *et al.*, 2017) and was based on the Nielsen’s panel. It analysed how different types of households (low/high income) and consumers (with healthy/unhealthy behaviours or diet) reacted to this tax, by implementing a pre–post study design (2012, prior to tax implementation, to 2015, post-tax implementation). The study reported that the total volume of taxed products purchased declined by 4% in 2014 and by 14.2% in 2015, while the untaxed purchase changes were higher in 2014 (+2.8%) but declined in 2015 (−4.9%). The household subgroup analysis reported that, in the post-tax implementation period (2014–15) compared with the pre-tax period (2012–13), the low-income household group consumption decreased by 1.3%, the high-income household consumption (i.e. those purchasing a lot of both taxed and untaxed products), decreased by 1.2%; consumers, whose consumption patterns were considered to be ‘unhealthy’ (i.e. consuming more taxed products and less untaxed products) decreased their total consumption by 4.9%, while consumers whose consumption patterns were considered to be ‘healthy’ (i.e. consuming more untaxed products and less taxed products) registered no differences in the post-tax period. Overall, the study reported a higher decrease in the second year after the implementation, compared with the first. The authors argue that this could be caused by many factors, probably by a gradual shift in consumer habits or by awareness campaigns on the harmful health impact of these products. The major gap between healthy and unhealthy households in consumption patterns might be explained by the fact that healthy consumers are already less inclined to buy harmful foods compared with those used to buy them. The study confirmed a trend of reduction in the consumption of energy-dense ultra-processed foods after tax implementation in Mexico.

Two studies (Caro *et al.*, 2018; Nakamura *et al.*, 2018) analysed the impact of the “Impuesto Adicional a las Bebidas Analcoh_**ò**_licas” (IABA) related to SSBs in Chile, which was implemented in October 2014. Specifically, in 2014, there was an increase in the tax rate from 13% to 18% on beverages with high levels of sugar (H-SSBs), defined as beverages with >6.25 g of sugar per 100 ml. Conversely, there was a tax decrease for beverages containing <6.25 g of sugar per 100 ml. Both studies showed a decrease of H-SSBs consumption in the post-increase period, compared with the pre-increase period. Caro *et al.* (2018) reported a monthly per capita decrease in H-SSBs purchases of 3.4% by volume, and 4% by calories, while the volume of L-SSBs increased of 10.7%, based on a post-increase period from November 2014 to December 2015 and a pre-increase period, as counterfactual, from 2013 to October 2014. Nakamura *et al.* (2018) also reported an H-SSBs monthly purchased decrease of 21.6%, by comparing the post-increase period (November 2014 to December 2015) to a pre-increase period that started in 2011. However, both studies agreed that the small increase in the SSB tax did not impact the population significantly, and that based on the small cohort observed and the short post-tax period it was not possible to assess the causal effect of the tax.

In addition to the research focusing on Mexico and Chile, another study (Claro *et al.*, 2012) attempted to evaluate price and income elasticity related to SSBs in Brazil. Although, strictly speaking, not a taxation study, the study simulated the effects on consumption of a 1% increase in price and 1% increase in income and analysed SSB taxation practices in Brazil; the study reported that a 1% increase in the price would cause a 0.85% reduction in SSBs product consumption. Additionally, changes in family income would influence SSBs consumption: for a 1% increase in family income there would be a corresponding 0.41% increase in SSBs consumption. Overall, poor households in Brazil would be more than twice as likely, relative to wealthy households, to change their consumption patterns if price and income changed. The study, however, underlined how these estimates were based only on home food and beverage consumption, approximately accounting for 76% of total household expenditure, leaving almost a quarter of purchasing patterns unaccounted for by the analysis.

#### Tobacco

Fifteen studies evaluated various aspects of tobacco use, i.e. the effect of tax implementation on consumer behaviour, the role of illicit tobacco product consumption, how price and income elasticity were shaped in each country and how elasticity could potentially change or was found to change following tax implementation. Mexico was included in four studies; the country dealt with a tobacco-related reform process which commenced after the ratification of the Framework Convention on Tobacco Control (FCTC) in 2004 and lasted for nearly a decade. Mexico is considered to be a country with a heavy burden of tobacco-related ill-health, reporting a smoking rate of 14.5% among Mexican adults (WHO, 2015). Three of the identified studies (Saenz-de-Miera *et al.*, 2010; Guerrero-Lopez *et al.*, 2013; Reynales-Shigematsu *et al.*, 2015) focused on the effect of the new tax structure (updated to 2011) on tobacco consumption levels, through country-level surveys and self-reported price of cigarettes by consumers. The research mainly underlined how smoking rates declined by 30% during 2002–15, how adolescent and adult groups reduced tobacco consumption in response to the specific excise tax introduction, and how the reform process uniformly affected all sociodemographic groups.

A narrative review on Argentina (Goodchild *et al.*, 2016) reported that tobacco affordability rose by 100% between 1997 and 2007, whilst the country experienced sharp economic growth. The study offered significant insights on how the introduction of an excise tax on tobacco would significantly reduce smoking prevalence (it was assumed that a 10% price increase would reduce the prevalence by 3%). Another study (Ferrante *et al.*, 2007) used a tobacco policy simulation model to evaluate how policies introduced in Argentina, relating to advertising, promotion and sponsorship bans, would have an effect on consumption. The study reported that these policies, regardless of the low level of taxes on cigarettes compared with HICs, produced a relative reduction in tobacco consumption in 2004 compared with 2001.

The literature also provides evidence on the extent of ‘illicit consumption’ of tobacco products and the effect of overall illicit smoking prevalence. Illicit consumption refers to consumption of tobacco products not legally purchased (e.g. counterfeit cigarettes). Three studies (Iglesias, 2016; Iglesias *et al.*, 2017; Szklo *et al.*, 2018), all from the Brazilian context, estimated how illicit cigarette consumption changed after the excise tax implementation in 2012, using national surveys (GATS-Brazil, Vigitel). The studies researched how the excise tax implementation impacted the overall proportion of illicit cigarette use among smoking population or on illicit smoking prevalence, looking at the general population or focusing on adults aged 18 years or older (see Table 2). All studies showed a reduction in smoking prevalence, but at the same time, an increase in illicit consumption from 16.9% in 2008 to 32.3% in 2013 was observed and continued to grow until 2016, when the estimated proportion of illicit consumption reached 42.8%. Curti *et al.* (2015) analysed whether a price increase in tobacco products would encourage smokers to consume cheaper tobacco products in Uruguay, by switching their consumption to illicit tobacco products. The study reported that a 10% price increase would increase by 4.6% the probability of consuming roll-your-own cigarettes over more expensive manufactured legal cigarettes, suggesting that it is relevant to narrow different tobacco product prices in order to successfully reduce overall consumption.

The last point of the tobacco consumption analysis is related to the price and income elasticity of demand, whether the demand for tobacco products is elastic or inelastic and whether tobacco products are normal and necessary goods. Data from five countries (Argentina, Colombia, Ecuador, Mexico, Peru) were identified and based on the evidence provided, both price and income were found to shape household or individual behaviour. Specifically, across all five countries, demand for tobacco products was found to be inelastic (with price elasticity of demand <−1, indicating low responsiveness to price changes; e.g. a 10% increase in the final price of tobacco products would result in a decrease in consumption by <10%). This could occur for various reasons, mainly related to consumer information on the new price, the level of addiction or lack of awareness of the risk related to tobacco products. In terms of the responsiveness of the demand for tobacco products to a change in income, captured by the income elasticity of demand, the evidence from all five countries showed that with an increase in income, tobacco consumption increased less than proportionally. The reported results confirmed that tobacco products are normal goods (income elasticity of demand being >0, with consumers raising consumption levels as their purchasing power increases) (Pindyck *et al.*, 2018); they were also found to be ‘necessities’ (income elasticity of demand being >0 but <1) (Table 3).

**Table 3:** Price elasticityand income elasticityof demand for cigarettes in select Latin American countries

Study name	Publication year	Study country	Price elasticity of demand^a^	Income elasticity of demand^b^
Chavéz	2016	Ecuador	−0.87	N/A
Gonzalez-Rozada *et al.*	2016	Peru	−0.70	0.112
Iglesias *et al.*	2017	Argentina	−0.28	0.41
Jiménez-Ruiz *et al.*	2008	Mexico	−0.52	0.49
Maldonado *et al.*	2016	Colombia	−0.78	0.61
Martinez *et al.*	2013	Argentina	−0.31	0.43

^a^ Demand is considered to be inelastic if for an increase in price by 1%, demand declines by <1%. In this case, the price elasticity of demand is negative and between −1 and 0.

^b^ If the income elasticity of demand is >0, this indicates a normal good; if the income elasticity of demand is <1 a good is a ‘necessity’, i.e. where income rises by 1% but demand for that good rises by <1%; if the income elasticity of demand is >1, then the good in question is a luxury.

*Source:* The authors from the literature.

#### Alcohol

Only one study (Chavéz, 2016) analysed alcohol consumption, and the effect of price elasticities of demand for tobacco and alcohol. The study reported a higher effect based on the price elasticity of demand for tobacco (−0.87) compared with alcohol (−0.44). The study also assessed the elasticity compared with Chilean total expenditure based on the quantity and quality of the goods, finding that the elasticity of alcohol consumption relating to total expenditure was 0.41 (compared with 0.5 for tobacco consumption), meaning that the variation in the quantity of consumed alcohol was relatively inelastic compared with the tobacco when total expenditure increased. If total expenditure declined, high-quality cigarettes and alcohol consumption would also decline, the latter being more sensitive to expenditure changes.

### Effect on revenue generation

#### Tobacco

Nearly all studies on revenue generation (9 out of 10) focused on revenues from tobacco taxation. Two studies approached this topic by considering multiple Latin American countries. One of them (Goodchild *et al.*, 2017) examined the effect of tax increases on weighted average prices, revenue generation and volume. On average, a 50% tobacco tax increase across the Latin American region would raise weighted average tobacco product prices by 28%, generate US$7 million revenue (+32%), and reduce the volume consumed by 7%; this trend would be traced in nearly all Latin American countries. The other study that considered the entire region (Garcés *et al.*, 2014) did not analyse a potential implementation but, rather, compared how Central American countries adapted to the FCTC directives. The analysis showed an overall gap that needed to be filled, due to the political and economic complexity of the area, and a lack in prioritization of research on legislation related to tobacco.

Six studies analysed the revenue effect of tobacco taxation at country level. Two of these (Iglesias, 2016; Iglesias *et al.*, 2017) studied how the implementation of two alternative taxation systems (either an ad valorem, or a mix of specific and ad valorem) allowing manufacturers of tobacco products to choose from in the Brazilian context impacted fiscal revenue and, as a consequence, changed levels of illicit consumption. In the Brazilian tobacco tax reform tobacco producers could choose between two regimes: a general regime, similar to the taxation system prevailing since 1999, where the ad valorem rate would have been 45% of the consumer price; and a special regime with a mix of specific and ad valorem rates. The latter has a lower ad valorem rate that could not be higher than 15%. Results were uniform in both studies: although revenue collection more than doubled in the observed period (2006–13) in absolute terms, sin tax introduction led to an increase in the illicit market, both in absolute terms and proportionally to the legal market (illicit daily tobacco consumption increased from 16.6% in 2008 to 31.1% in 2013). Based on that, the study concluded that it would be possible to increase revenue from taxation, despite the increase in the illicit market. A simulation study (Jimenez-Ruiz *et al.*, 2008) estimated that, with other factors being constant, a 10% price increase of tobacco products would yield an increase in revenue by 15.7% in Mexico. Another study (Rodriguez-Iglesias *et al.*, 2017) reported that despite the changes in real income and the final prices of cigarettes, even a 100% price increase in a low-revenue scenario would be beneficial for revenues and sustainable for the market. A study sampled 15 countries (including Brazil, Mexico and Uruguay from Latin America) to analyse the range of prices paid for cigarettes (Kostova *et al.*, 2014) and suggested that a uniform high excise tax would be more likely to reduce the range of cigarette prices compared with a tiered tax structure (i.e. where cheaper cigarettes are taxed at a lower rates than more expensive cigarettes) in each of the study countries, all of which were LMICs. Levels of excise tax are one the main components of tobacco prices and price ranges of tobacco products can determine purchase levels. Bardach *et al.* (2016) adopted a micro-simulation model to assess, among other things, smoking impact on costs associated with a set of cardiovascular, pulmonary and oncology diseases and found that, with a 50% price increase of tobacco products, Peru would collect 3.14 billion of Peruvian Sol (equivalent to US$1.05 billion) in the 10 years following the price increase. Finally, a study (James *et al.*, 2019) researched how a tax increase in Colombia could potentially impact revenue generation. The tax increase, legislated in December 2016, tripled the specific excise taxes and increased VAT by 3%, leading to a 70% relative price increase in tobacco products. Based on a simulation and following the introduction of the new increases, the net annual gains in tax revenue were estimated at COP$1.26 billion (approximately US$364 million) compared with the pre-tax net annual gains (2016) over a 20-year period.

#### Sugar-sweetened beverages

The only study (Sánchez-Romero *et al.*, 2016) addressing the effect of a nationwide SSB tax on consumption simulated how a potential reduction in SSB intake, following a tax increase, beyond revenue generation, would impact on direct diabetes healthcare costs in Mexico in terms of generating potential healthcare cost savings. The simulation was based on two different scenarios, notably a 10% and a 20% reduction in SSB consumption, also taking into account any potential replacement for calorie compensation. Simulation results reported that, with a 10% reduction in SSB consumption, 983 million international dollars would have been saved over a period of 9 years, while a 20% reduction would have led to a saving of 1.9 billion international dollars.

### Impact on health improvement

#### Sugar-sweetened beverages

The only included study for this endpoint analysed the sin tax impact on health in Mexico (Sánchez-Romero *et al.*, 2016). The Mexican population suffers from high rates of diabetes, excess weight and obesity, and cardiometabolic problems, all of which are strongly associated with increased SSB intake (Sánchez-Romero *et al*., 2016). In order to quantify how excise taxes on SSBs could lead to changes in health outcomes, Sánchez-Romero *et al.* (2016) simulated the effects of two scenarios, a 10% and a 20% reduction in SSB consumption, both with a 39% calorie compensation (i.e. still receiving 39% of daily calorie intake through non-SSB foods or drinks), and their impact after 10 years. Results in both scenarios showed a significant reduction in the number of people affected by diabetes, suffering a stroke or a heart attack and an overall reduction in deaths, particularly in the 35–49 age group.

#### Tobacco

The impact of tobacco on health outcomes was addressed by five studies.

A study on Peru (Bardach *et al.*, 2016) estimated that in 2015, 31% of all deaths (∼16,833 out of 54,301) in the country were associated with tobacco consumption. The study calculated that a 25% price increase in tobacco through taxation could reduce the number of deaths by 6,695 over a period of 10 years; a 50% price increase would potentially avoid 13,391 deaths, while a 100% price increase would avoid 26,782 deaths over 10 years. A study on Argentina (Ferrante *et al.*, 2007) developed a simulation model to assess how tax increases in tobacco retail prices would impact avoidable deaths. Two tax increase scenarios were adopted: one at 75% (compared with taxation at 68% in 2007, leading to an overall 28% price increase) and one at 85% (with a final price increase of 113%). With a 75% increase, 1,899 deaths per year would be avoided over a 20-year period (2004–24), and a further 2,911 deaths would be prevented in the 2024–34 period. With an 85% increase, 7,581 deaths per year would be avoided until 2034. In the context of Mexico, despite the ratification of FCTC, the number of deaths associated with tobacco consumption increased from 47,800 to 56,800 in the 2002–13 period (Reynales-Shigematsu *et al.*, 2015). Through the use of the SimSmoke model, it was estimated that the implemented policies in Mexico (taxation, health warnings, smoke-free air laws, advertising restrictions), would prevent 3,000 deaths in 2013, and contribute to an overall reduction in the death rate by 10,800 in the 2002–13 period. Additionally, the model predicted that the current regulation would prevent 826,000 smoking-related deaths by 2053. Smoking ban regulation and tobacco tax increase were tested by a study (Jan *et al.*, 2014) for association with the risk of having an acute myocardial infarction (AMI) in Panama. The smoking ban was issued in May 2008 while the tax increase was implemented in November 2009. The study set two pre-tax periods (May 2008 to April 2009 and May 2009 to November 2009) and a post-tax period (December 2009 to December 2010) of intervention as periods of observation and was based on hospital admission data. Results showed that the relative risk of having an AMI was similar in all three periods (first period: 0.982; second period: 1.049; third period: 0.985), underlining how these two policies had no short-term effect on CVD prevalence. A micro-simulation model set in Peru estimated that a 50% price increase in tobacco products would avoid nearly 14,000 deaths, 6,210 cardiovascular events and 5,361 new cancer cases over a period of 10 years (Bardach *et al.*, 2016). Finally, evidence from Colombia (James *et al.*, 2019), simulating whether the 2016 average price increase in cigarettes might result in additional life-years gained (LYG), found that over a period of 20 years the impact would be 191,000 additional LYG, of which 50% would come from the two lowest income quintiles and only 28% from the the highest income quintile.

### Risk of bias assessment results

Table 4 shows the low, medium, high and unclear risk of bias occurring in each domain and categorizes high risk of bias in sub-categories. Each sub-category has a number that is included in the risk of bias table and represents the specific type of risk of bias. Due to the nature of the included studies the ROBINS-I tool was adopted, specifically designed to assess risk of bias in non-randomized studies. Twenty-eight out of 34 studies reported at least a medium/unclear or high risk of bias in at least one of the seven dimensions we have considered (confounding; selection of participants; intervention classification; deviation from intended intervention; missing data; outcome measurement; and selection of reported results). Most of the medium/high risk of bias were related to the outcome measurement (13 studies reported high risk, while 5 reported medium/unclear risk), followed by missing data (10 studies reported high risk, 2 reported medium/unclear) and deviation from intended intervention (9 studies reported high risk while 2 reported medium/unclear). Conversely, only 2 studies reported risk of confounding bias (1 high risk and 1 medium/unclear), and 3 reported intervention classification bias (0 high risk and 3 medium/unclear). Results showed a relevant presence of moderate or high risk of bias specifically in the missing data and the outcome of measurement domains. Missing data bias was primarily due to the lack of information on geographical coverage, production chain (manufacturer or retailer data), economic and social indicators. Bias in outcome measurements, due to self-reported data and underestimation of intervention and/or comparators, were often linked to a vague composition of data. A more detailed description of risk of bias is available in Table 4 (and more detailed information is provided in Supplementary AppendixTable SA1).

**Table 4:**
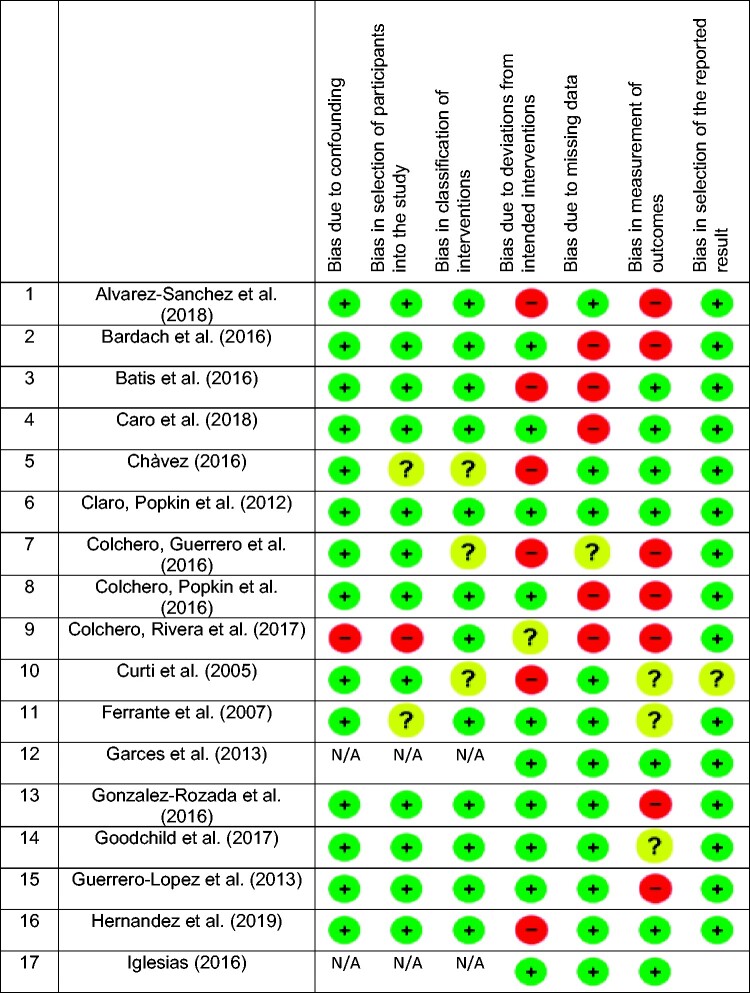

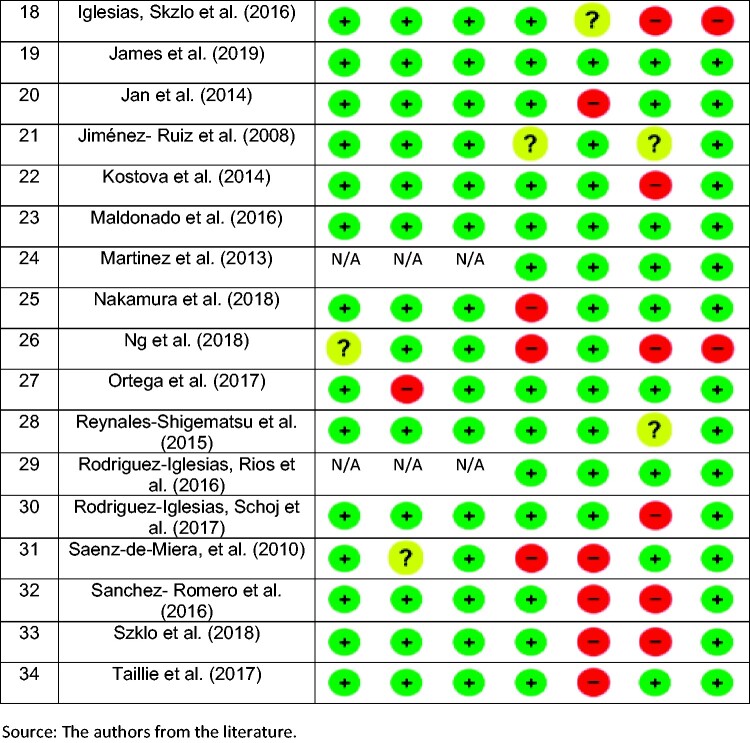
Sin taxes in the Latin American context: summary of risk of bias assessment

The table above summarizes the risk of bias level of each study. Green, yellow and red dots, respectively, indicate low, moderate/unclear and high risk of bias according to each domain.

*Source:* The authors from the literature.

## Discussion

This SLR identified and assessed the impact of sin taxes on goods that are considered to be harmful from a public health perspective in Latin American countries from 2000 to 2018, by analysing the evidence based on three endpoints: effect on consumption, effect on revenue and health impact and is the first that is doing so in the Latin American region. Twenty-three out of 27 studies examining consumption effects confirmed that the application of a sin tax was inversely related to consumption levels. In the case of SSB tax in Mexico and its effect on consumption, this has been analysed by seven studies, six of them confirming the inverse relationship between tax introduction and consumption levels. Evidence from 10 studies analysing the revenue endpoint is aligned in supporting excise tax implementation or increase in the region to support additional revenue generation in a sustainable manner, providing, among others, case studies focused on Argentina, Brazil, Colombia, Mexico and Peru. Finally, five out of six studies focusing on the likely impact on health showed through a series of simulation models that potential sin tax implementation or increase would avert thousands of deaths, particularly from CVD and cancer, as well as lead to hundreds of thousands of additional LYG in a relatively short timeframe. Table 5 provides a summary of sin tax effect(s) or impact(s) and the extent of the effect(s) or impact(s) reported by each study. None of the studies reported a negative effect or impact on any of the three endpoints.

**Table 5:** Sin tax implementation/increase and effects on consumption, revenue generation and health: summary results from the literature

First author(s)	Other endpoint(s)	Tax/indicator of interest	Setting	**Tax effect/ impact (+/-)** ^a^	Extent of the impact
Consumption					
Álvarez-Sánchez *et al.* (2018)	No	SSBs	Mexico	Positive	People’s awareness of sin tax implementation and decrease in consumption were directly proportional, i.e. people who were aware of the tax introduction were more inclined to decrease their SSB intake
Batis *et al.* (2016)	No	SSBs	Mexico	Positive	Household purchases of non-essential energy-dense foods declined in the first year after SSBs and non-essential food taxes in Mexico
Caro *et al.* (2018)	No	SSBs	Chile	Mildly positive	SSBs tax in Chile has mildly increased prices of H-SSBs (i.e. higher-taxed SSBs). Carbonated H-SSBs increased by 2%, while non-carbonated H-SSBs increased by 3.9%. Household monthly per capita purchases of H-SSBs decreased by 3.4% by volume and 4% by calories, with a greater change among high socioeconomic status households. Modifications of Chile's SSB tax were small, and so its impact on beverages purchases.
Chavéz (2016)	No	Demand for tobacco	Ecuador	Positive	Price elasticity of demand for cigarettes is −0.87 in Ecuador. Results for cross-price elasticities of alcohol on cigarette demand are negative, as expected, indicating that they are complementary goods; however, study results on cross-price elasticity are not statistically significant. Policy of price increases applied to both cigarettes and alcohol could reduce their consumption.
Claro *et al.* (2012)	No	SSBs	Brazil	Positive	1% increase in SSBs price led to a 0.85% reduction of SSB calories consumed in Brazil. High SSB price elasticity indicates that on SSB (both on purchased weight or volume) leads to a decrease in SSB consumption
Colchero *et al.* (2016s)	No	SSBs	Mexico	Positive	SSBs tax in Mexico led to a decrease of 7.3% in per capita sales of SSBs and at the same time to an increase of 5.2% of per capita sales of plain water compared with the pre-tax period. SSB tax is associated with a reduction in per capita sales of SSB
Colchero *et al.* (2016b)	No	SSBs	Mexico	Positive	After 1 year of SSBs tax implementation purchases of taxed beverages decreased on average by 6% (compared with the counterfactual), with all the socioeconomic groups reducing their purchases levels. Study reports an association between SSB tax implementation, reductions in taxed beverages purchases and increases in untaxed beverages purchases.
Colchero *et al.* (2017)	No	SSBs	Mexico	Positive	SSBs tax led to a decrease in taxed beverages purchases of 5.5% in 2014 and 9.7% in 2015 compared with the pre-tax period (2012–13). Findings support the association between SSB tax implementation and decrease in taxed beverages purchases.
Curti *et al.* (2015)	No	Tobacco excise tax	Uruguay	Neither positive nor negative	Ten per cent price increase of tobacco prices would increase by 4.6% the probability of consuming roll-your-own cigarettes over more expensive manufactured legal cigarettes, suggesting that it is relevant to narrow different tobacco products prices in order to successfully reduce overall consumption
Ferrante *et al.* (2007)	Health	Tobacco excise tax	Argentina	Neither positive nor negative	Policies like advertising, promotion and sponsorship bans have an effect on consumption even in a context like Argentina where taxes on tobacco are relatively low. The study reports how excise tax implementation can benefit from a wider set of related policies to be more effective
Gonzalez-Rozada and Ramos-Carbajales (2016)	No	Tobacco	Peru	Positive	Demand price elasticity was −0.7%, meaning that a 10% price increase via a new tax would reduce consumption by 7%. Study reports how increasing excise taxes to tobacco products is one of the best options to reduce tobacco consumption
Guerrero-Lopez *et al.* (2013)	No	Tobacco	Mexico	Positive	Study reports how tobacco consumption declined after tobacco tax implementation in 2012, while between 2000 and 2012 there were no changes in smoking prevalence
Hernández *et al.* (2019)	No	8% tax on energy-dense nutrient-poor foods	Mexico	Positive	Following the implementation of the energy-dense nutrient poor-foods tax there was a decrease of 5.4 g/week per capita in taxed food consumption compared with pre-tax period (2008, 2010 and 2012). The study reports how tax implementation has been effective in reducing taxed food purchases.
Iglesias (2016)	Revenue	2011 tobacco tax	Brazil	Positive	Tobacco tax and price increases reduced total cigarette consumption. Smoking prevalence dropped from over 13% in 2011 to 10.8% in 2014.
Iglesias *et al.* (2016)	Revenue	2012 cigarette excise tax	Brazil	Positive	Cigarette tax increase reduced smoking prevalence. Daily manufactured cigarette smoking prevalence rates moved from 13.3% in 2008 to 10.8% in 2013. However, illicit manufactured consumption moved from 16.6% to 31.1% in the same period.
Jiménez-Ruiz *et al.* (2008)	Revenue	VAT (Tobacco)	Mexico	Positive	Total price elasticity is −0.52, meaning that a 10% in the cigarette price would lead to a −5.2% decrease in average cigarette consumption. Higher prices would reduce household smoking largely in terms of smoking prevalence.
Maldonado *et al.* (2016)	No	Tobacco	Colombia	Positive	Tobacco demand is sensitive to price and income. Demand price elasticity is −0.78, while income elasticity is 0.61. Study supports a higher taxation on tobacco to reduce consumption in the country.
Martinez *et al.* (2015)	No	Tobacco tax	Argentina	Positive	Long-term income elasticity was 0.43, while own-price elasticity was equal to −0.31, meaning that a 10% increase in the growth of real income led to an increase of tobacco consumption of 4.3%, while a 10% price increase produced a fall of 3.1% in cigarette consumption. Short-term income elasticity was 0.25, while short-term own-price elasticity of cigarette demand was −0.15. Study suggests how findings on elasticity provide positive evidence on tobacco tax increases.
Nakamura *et al.* (2018)	No	SSBs	Chile	Mildly positive	After tax implementation, there was a significant decrease in the monthly purchased volume of higher-taxed sugary soft drinks by 21.6%. Reduction in soft drink purchasing was higher in higher socioeconomic groups and in higher pre-tax purchasers of SSBs. However, evaluation did not involve a randomized design, therefore results cannot demonstrate a causal inference.
Ng *et al.* (2019)	No	SSBs	Mexico	Positive	After SSBs tax implementation, there has been a reduction in consumption of SSBs, particularly among high SSBs purchasers, compared with the pre-tax period.
Ortega-Avila *et al.* (2018)	No	SSBs	Mexico	Neither positive nor negative	Limited awareness among adolescents of the SSB tax implementation. Impact of the tax could be misperceived by some segments of the population and that this would represent a limitation in changing citizens’ attitudes towards these products.
Reynales-Shigematsu *et al.* (2015)	Health	Tobacco tax	Mexico	Positive	Tobacco consumption gradually decreased in the 2002–13 period as a result of policies implemented since 2002 (FCTC ratification in 2002, tobacco tax increases from 2007 to 2011).
Rodriguez-Iglesias *et al.* (2016)	No	Tobacco	Argentina	Positive	Cigarette tax increase that raises prices by 10% would lead to a decrease in smoking consumption of 3%. Authors suggest pursuing this policy option as price of cigarettes in the country is one of the lowest in the world.
Rodriguez-Iglesias *et al.* (2017)	Revenue	Tobacco	Argentina	Positive	Long-term price elasticity is −0.279, meaning that a 10% increase in real prices reduces cigarette consumption by 2.79% per quarter. Long-term income elasticity is 0.411, meaning that a 10% increase in real income raises consumption by 4.11% per quarter. There is wide margin for excise tax implementation in the country in order to effectively reduce consumption
Saenz-de-Miera *et al.* (2010)	No	SPST and VAT	Mexico	Mildly positive	2007 VAT tax increase, although indirectly, likely led to a general decrease in tobacco consumption.
Szklo *et al.* (2018)	No	Illicit tobacco consumption	Brazil	Neither positive nor negative	Illicit cigarette use increased in Brazil, both overall and across two socioeconomic groups of smokers who did not stop smoking, after a new cigarette excise tax implementation. Illicit consumption needs to be carefully considered as a potential consequence of excise tax increase or implementation.
Taillie (2017)	No	SSBs	Mexico	Positive	Total volume of taxed products purchased declined by 4% in 2014 and by 14.2% in 2015, while the untaxed purchase changes were higher in 2014 (+2.8%) but declined in 2015 (−4.9%). The study confirmed a trend of reduction in the consumption of energy-dense ultra-processed foods following tax implementation.
Revenue					

Bardach *et al.* (2016)	Health	Tobacco	Peru	Positive	Model results report that, with a 50% price increase of tobacco products, Peru would collect 3.145 billion of Peruvian Sol (approximately US$ 1.05 billion) in the 10 years following price increase.
Garcés *et al.* (2014)	No	Tobacco	Central America	Positive	Excise taxes implementation or increase on tobacco products is encouraged in Latin America, as tobacco taxes in the region are overall far lower than the levels recommended by FCTC.
Goodchild *et al.* (2017)	No	Tobacco excise taxes	Latin America and Caribbean	Positive	A 50% tobacco tax increase across the Latin American region would raise weighted average tobacco product prices by 28%, generate US$ 7 million revenue (+32%), and reduce the volume consumed by 7%. This trend would be traced in nearly all Latin American countries.
Iglesias (2016)	Consumption	Tobacco tax	Brazil	Positive	2011 reform increased revenues by 20% compared with pre-tax year, despite legal sale contraction. The reform succeeded in revert the previous policies of low taxation on tobacco products, generating revenues for the country.
Iglesias *et al.* (2016)	Consumption	2012 cigarette excise tax	Brazil	Positive	Raising excise taxes increased government revenues. Cigarette excise tax collection doubled between 2006 and 2013, from US$1.10 billion to US$2.36 billion. However, illicit tobacco trade increased.
Kostova *et al.* (2014)	No	Tobacco	Brazil, Mexico, Uruguay	Neither positive nor negative	The study suggests that a uniform high excise tax is more likely to reduce range of cigarette prices compared with a tiered tax structure (i.e. where cheaper cigarettes are taxed at a lower rates than more expensive cigarettes) in each of the 15 considered countries (all LMICs). Levels of excise taxes are one the main components of tobacco prices.
James *et al.* (2019)	Health	Tobacco tax	Colombia	Positive	Tax increase was legislated in December 2016, tripled the specific excise taxes and increased VAT by 3%, leading to a 70% relative price increase in tobacco products. The study simulated that, after a full introduction of the new increases, in 20 years the net annual gains in tax revenue would be of COP$1.26 billion (approximately US$364 m) compared with the pre-tax net annual gains (2016).
Jiménez-Ruiz *et al.* (2008)	Consumption	VAT	Mexico	Positive	Study simulated that, holding other factors constant, a 10% price increase of tobacco products would yield a rise in revenue by 15.7%, providing strong arguments for increasing cigarette taxes
Rodriguez-Iglesias *et al.* (2017)	Consumption	Tobacco	Argentina	Positive	Study simulated that, even in a conservative scenario, despite the changes in real income and the final prices of cigarettes even a 100% price increase in a low-revenue scenario would be beneficial for revenues and sustainable for the market.
Sánchez-Romero *et al.* (2016)	Revenue	SSBs	Mexico	Positive	Simulation was based on two different scenarios, i.e. a 10% and a 20% reduction in SSB consumption, and by taking into account any potential replacement for calorie compensation. Simulation results reported that, with a 10% reduction 983 million international dollars would have been saved in a time span of 9 years, while a 20% reduction would have led to a saving of 1.9 billion international dollars.

Health					

Bardach *et al.* (2016)	Revenue	Tobacco	Peru	Positive	Micro-simulation model estimated that a 50% price increase of tobacco products would lead in the next 10 years to almost 14 000 avoided deaths, 6210 avoided cardiovascular events and 5361 avoided new cancer cases.
Ferrante *et al.* (2007)	Consumption	Tobacco excise tax	Argentina	Positive	Developed a simulation model to assess how tax increases in the retail price of tobacco would impact avoidable deaths. Two tax increase scenarios on the retail price were adopted: one at 75% (compared with taxation at 68% in 2007, leading to an overall 28% price increase) and one at 85% (with a final price increase of 113%). With a 75% increase, 1899 deaths per year would be avoided over a 20-year period (2004–24), and 2911 deaths would be prevented in the 2024–34 period. With an 85% increase, 7581 deaths per year would be avoided until 2034.
James *et al.* (2019)	Revenue	Tobacco tax	Colombia	Positive	Simulated how the 2016 average price increase on cigarettes would result in additional LYG. In 20 years, the authors expected about 191 000 LYG, of which 50% would come from the two groups with poorest income quintiles, and only 28% from the group with the highest-income quintiles.
Jan *et al.* (2014)	No	TTI	Panama	Positive	Tobacco tax increase reduced AMI in the 2006–10 period. First year of comprehensive smoking ban policies (including tax increase) was associated with an acute myocardial infarct relative risk reduction of 1.8% (adjusted Poisson regression model).
Reynales-Shigematsu *et al.* (2015)	Consumption	Tobacco tax	Mexico	Positive	Estimated with SimSmoke model that the implemented policies in Mexico (taxation, health warnings, smoke-free air laws, advertising restrictions), contributed to avoid 3000 deaths in 2013, and an overall reduction in the death rate by 10 800 in the 2002–13 period. Additionally, the model predicted that the current regulation would prevent 826 000 smoking-related deaths by 2053.
Sánchez-Romero *et al.* (2016)	Health	SSBs	Mexico	Positive	Simulated the effects of two scenarios, a 10% and a 20% reduction in SSB consumption, both with a 39% calorie compensation (i.e. still receiving 39% of daily calorie intake through non-SSB foods or drinks), and their impact after 10 years. Results in both scenarios showed a significant reduction in the number of people affected by diabetes, suffering a stroke or a heart attack and an overall reduction in deaths, particularly in the 35–49 age group.

a A positive effect in the *consumption section* means that there is a decrease in consumption after tax implementation, while a negative effect means that no decrease in consumption is detected. With regards to the *health and revenue sections*, a positive effect means that there are health improvements or new revenues with tax implementation and vice versa.

*Source:* The authors from the literature.

Results and conclusions on the association between sin tax implementation or increase and decrease in the consumption of harmful goods for public health, improved population health conditions or new sources of revenue in Latin America are aligned and compatible with findings from the literature in other geographical areas. An earlier systematic review (Wright *et al.*, 2017) with different criteria analysing 102 studies, focused on how consumption levels and revenue generation could be affected by public health taxes. This review did not focus on a specific geographical area, but the vast majority of the studies included came from HICs. Nevertheless, it confirmed the effectiveness of sin taxes as a tool for reducing harmful goods consumption, while revenue collection would be dependent on a variety of factors, e.g. the effectiveness of taxation in changing behaviour. Another recent systematic review (Redondo *et al.*, 2018) has analysed results from 17 studies examining how the impact of taxes could shape SSB consumption. Likewise, the inverse relationship between SSB consumption and taxation levels was confirmed. Our study reinforces all these findings particularly with regards to the decrease in consumption and, additionally, expands the research rationale by investigating the potential association between sin tax introduction and likely health outcomes.

However, our study also portrayed a very complex context in which the policy-making process faced many obstacles to achieve the ideal tax reforms required for this purpose. Latin America consists primarily of middle- and upper middle-income countries, with significant consumption of sugar, alcohol and tobacco. Despite high rates of tobacco consumption, tobacco taxation is generally underutilized compared with taxation levels in HICs (Sandoval *et al.*, 2016). Retrospective analysis of sin tax introduction and simulations confirmed that the current level of taxation in the region could be increased considerably and this could lead to a sustainable generation of FS. In this sense, countries in the region could effectively pursue one or more of the ways proposed in the FS analytical framework, e.g. introduce or raise taxation levels whilst also trying to improve healthcare efficiency. The extent to which sin taxes can successfully fund health care depends on many factors, including the type of sin tax, the response of consumption to price increases, captured by the price elasticity of demand, income levels, the burden of disease, the extent to which relevant taxes are hypothecated (earmarked) and, interestingly, the broader political consensus among stakeholders on choices related to public expenditure (Clements and Gupta, 2012), which, in turn shapes the political feasibility of introducing additional taxes. Lack of consensus has been showcased as an important factor in the Argentinian context, where the lobbying power of tobacco producers has diverted the government from adopting the measures included in the FCTC despite wide smoking prevalence in the country and the elevated burden of disease directly or indirectly attributable to tobacco (Mejia *et al.*, 2008). Argentina, with one of the lowest tobacco prices in the world (Rodriguez-Iglesias *et al.*, 2018) also experienced an increase in affordability over the last decade. Brazil is the third major producer of tobacco in the world (Gigliotti *et al.*, 2014), and is also facing extensive levels of tobacco lobbying. This can cause tensions among stakeholders and influence, or even shape, taxation policy.

Many of the included studies explicitly reported how even a strong tax increase in some products that are classed as (potentially) harmful would lead to a rise in total tax revenue, therefore, it would be an efficient way to raise revenue. However, it has also emerged that in some cases, particularly as concerns tobacco and alcohol, an increase in taxation would not automatically generate a certain amount of revenue, since levels of consumption might be different from expected or because the illicit market could grow and replace the legal market, at least in part. Consequently, there are broader considerations shaping the discussion around the introduction of sin taxes, in this case, law enforcement to counter the effects of illicit trade. On the other hand, the long-term health consequences of continued consumption of tobacco, alcohol or sugary drinks can be considerable. Countries like Mexico face significant health challenges related to diabetes, with the highest prevalence among OECD countries (Levy *et al.*, 2018), obesity and CVD, some of which is attributable to high consumption of SSBs over long periods of time.

Guidelines from inter-governmental organizations on sin tax implementation have been only partially followed by Latin American countries. The WHO FCTC (2003) and the MPOWER Report (2008) state that an increase in taxes on cigarettes, country promotion of bans on advertising, laws on smoke-free areas, health warnings, media campaigns and policies for treatment cessations, if applied in a systematic way, would significantly reduce tobacco consumption rates in adults (Paoletti *et al.*, 2012; Levy *et al.*, 2018). In particular, article 6 of the FCTC reports that the increase in tobacco price through excise taxation is the most cost-effective single measure in order to reduce the demand for tobacco and contribute smoking cessation improvement (WHO, 2003). These international guidelines are interfacing with a complex regional scenario, which is characterized by a particularly challenging epidemiological reality, significant levels of production of alcohol, tobacco and sugar and a timid political consensus over guidelines such as the ones by WHO in some countries.

The consumption level of harmful goods, the related burden of disease and the difficulties in the tax structure reform in Mexico were a clear example of how consumer habits, state of health and state regulation can have significant impact on outcomes in the health of the population and in the long-term sustainability of the health system. At the same time, even with a partial reform compared with what FCTC recommended, evidence from Mexico showed how a wider approach that included taxation and an organized set of other measures, could lead to a sensible improvement in all the endpoints considered.

The role of data collection and research related to sin taxes and their impact represented another relevant point which emerged from our study. Funded studies included in our systematic literature review received grants only from government organizations [e.g. National Institutes of Health (USA), the Brazilian Ministry of Health], international organizations (e.g. the World Bank), non-Latin American non-governmental organizations (e.g. Bloomberg Philanthropies) or academic institutions (e.g. University of South Carolina). Of course, in many countries, general research may be conducted by manufacturing industries (Chavéz, 2016; Iglesias, 2016) and, as such, could represent a source of bias as it represents corporate interests. Academic research leveraging country-level data appears to be limited as the only relevant sources are national surveys, often not including rural areas or relying on self-reporting methods. This creates a high risk of bias for researchers and policy-makers and has been already highlighted with regards to beverage industry statistics, which can be misleading and ‘fail to account for population or economic growth’ (Colchero *et al.*, 2017). The same was observed in the case of tobacco and how industry lobbying activities can strongly influence policy-making. Studies exist discussing how the tobacco industry concurred with the non-implementation of tobacco taxes in many parts of the world, despite the robust scientific evidence supporting their implementation (Jha and Chaloupka, 2000). The case of Argentina may represent the most telling example in Latin America of the relationship between the state and the tobacco industry that is weighted in favour of manufacturers’ aims.

For these reasons, the implementation of sin taxes varies across settings based on the specific targeting of goods, the effective amount of tax, the choice between ‘per unit’ vs ‘ad valorem’ taxes, and the use of potential FS created beside the underlying broader rationale that justifies sin tax implementation. Research has emphasized that the specific country framework with regards to overall health state, socioeconomic composition, consumer habits, policy-making processes and orientations determines the most effective pathway for a successful sin tax implementation in the case of SSBs (Brownell *et al.*, 2009; Claro *et al.*, 2012). That said, a specific definition of what products are targeted is necessary to avoid side-effects in consumption, such as provoking the use of other similar harmful goods (i.e. goods of dubious quality that might not be captured by the tax reform, such as low-quality foods or beverages).

The definition of the appropriate amount of tax is another controversial decision. Research on cardiovascular risk in young adults (Duffey *et al.*, 2010) concluded that only high rates of taxation would produce a significant change in consumption; this is consistent with recommendations made by many of the studies in this study.

The decision of adopting a ‘per unit’ vs ‘ad valorem’ tax is usually at the forefront of the debate. A per-unit sin tax is easier and more flexible to implement from a government regulation perspective than an ad valorem tax; generally, LMICs are encouraged to implement per-unit taxes because of limitations in law enforcement or administrative capacity. The disadvantages of this type of tax relate to the frequency and timing of revisions in order to ensure the tax remains effective. In fact, manufacturers can try to adjust the burden of the tax on some segments of the production process and, through that, reduce the price increase of the product to the consumer. At the same time, consumers can shift their consumption to lower-priced goods or to other similar products, keeping in mind, however, that for some products (e.g. tobacco and alcohol) substitution may be difficult. An ad valorem tax has its advantages because it easily adjusts to inflation changes, it is more visible and directly payable to the authorities.

Notwithstanding the discussion on the relative merits of per unit or ad valorem taxes, the predicted revenue from the tax is uncertain and requires careful monitoring. An analysis of European Union countries has demonstrated that per-unit taxes have a better yield than ad valorem taxes on retail cigarette prices (Delipalla and O'Donnell, 1998; Goodchild *et al.*, 2017). However, the overall preference of one tax over the other depends on specific country features and the specific objectives of policy-makers. Other studies (Wright *et al.*, 2017; Whitehead *et al.*, 2018) suggest that specific taxes would be more effective in reducing the consumption of certain goods since an ad valorem tax could potentially shift consumption to cheaper and lower-quality goods, or induce manufacturers to reduce prices in order to maintain consumption levels. A final, broader, point of discussion relates to the implications of sin tax introduction on choice and on its regressive nature. Two studies (Brownell *et al.*, 2009; Claro *et al.*, 2012) claim that the benefits of sin taxes, especially on health outcomes, outweigh their dis-benefit on choice. With regards to their regressive nature, products subject to sin taxes, such as cigarettes, tend to cause greater harm to lower socioeconomic groups, and although the latter are impacted financially more heavily than higher socioeconomic groups, the incentive to behavioural change is greater.

### Study limitations

The results of this study reflect Latin American countries’ economic, political and epidemiological features and reality; therefore, the results may not be generalizable to other geographical regions. Furthermore, the analysis compared countries with significant differences in regulation, epidemiological frameworks and economic conditions, while many studies analysed policy changes in just a few countries. Additionally, most studies analysed sin tax impact within a relatively short space of time and lack long-term evidence. Despite all the above, the broader results of this study are consistent with most of the recent academic literature and underline many potential benefits of sin tax implementation in middle-income countries.

## Conclusion

This study has confirmed the role of sin taxes in the Latin American context as a valid policy option for reducing consumption of harmful goods, generating additional revenue and potentially improving health outcomes. The majority of studies reported that implementation of sin taxes in Latin America resulted in reductions in harmful goods consumption, increases in revenue generation and a positive—albeit simulated—effect on health outcomes. The results on the risk of bias assessment and the analysis of the included studies suggested that future work on this topic would require more accurate data collection processes that go beyond weak study designs that may be susceptible to high risk of bias. This would require an increase in efforts to promote research and address stakeholder interests. Apart from improving data collection, a broader general effort is necessary in producing research on this topic; Latin American countries are gradually investing more in health and are aware of the costs associated with tobacco, alcohol and sugary beverages, but are still far from reaching HIC levels in terms of investment in health and tax intervention to mitigate the negative effects of these products.

## Supplementary data

Supplementary data are available at *Health Policy and Planning* online.

*Conflict of interest statement*. None declared.

*Ethical approval.* No ethical approval was required for this study.

*Acknowledgements*. We are grateful to the comments and suggestions of two anonymous referees. All outstanding errors are our own.

## Supplementary Material

czaa168_Supp

## References

[czaa168-B1] Álvarez-Sánchez C , Contento I, Jiménez-Aguilar A et al 2018. Does the Mexican sugar-sweetened beverage tax have a signaling effect? ENSANUT 2016. PLoS One 13: e0199337. 30133438 10.1371/journal.pone.0199337 PMC6104929

[czaa168-B2] Arsenault B , Lamarche B, Després J-P. 2017. Targeting overconsumption of sugar-sweetened beverages vs. overall poor diet quality for cardiometabolic diseases risk prevention: place your bets!. Nutrients 9: 600. 10.3390/nu9060600 PMC5490579 28608806

[czaa168-B3] Bardach A , Caporale J, Alcaraz A et al 2016. Carga de enfermedad por tabaquismo e impacto potencial del incremento de precios de cigarrillos en el Perú. Revista Peruana de Medicina Experimental y Salud Pública 33: 651. 28327833 10.17843/rpmesp.2016.334.2548

[czaa168-B5] Batis C , Rivera JA, Popkin BM, Taillie LS. 2016. First-year evaluation of Mexico’s tax on nonessential energy-dense foods: an observational study. PLoS Medicine 13: e1002057. 27379797 10.1371/journal.pmed.1002057 PMC4933356

[czaa168-B6] Brownell J , Farley T, Willet W et al 2009. The public health and economic benefits of taxing sugar-sweetened beverages. New England Journal of Medicine 361: 1599–605. 10.1056/NEJMhpr0905723 PMC3140416 19759377

[czaa168-B7] Caro JC , Corvalán C, Reyes M et al 2018. Chile’s 2014 sugar-sweetened beverage tax and changes in prices and purchases of sugar-sweetened beverages: an observational study in an urban environment. PLoS Medicine 15: e1002597. 29969444 10.1371/journal.pmed.1002597 PMC6029755

[czaa168-B8] Chavéz R. 2016. Price elasticity of demand for cigarettes and alcohol in Ecuador, based on household data. Pan American Journal of Public Health **6** :222–8. 28001197

[czaa168-B9] Claro RM , Levy RB, Popkin BM, Monteiro CA. 2012. Sugar-sweetened beverage taxes in Brazil. American Journal of Public Health 102: 178–83. 22095333 10.2105/AJPH.2011.300313 PMC3490548

[czaa168-B10] Clements BCD , Gupta S. 2012. The Economics of Public Health Care Reform in Advanced and Emerging Economies. Washington, DC: International Monetary Fund.

[czaa168-B11] Cnossen S. 2005. Theory and Practice of Excise Taxation: Smoking, Drinking, Gambling, Polluting, and Driving. Oxford: Oxford University Press.

[czaa168-B12] Colchero MA , Guerrero-López CM, Molina M, Rivera JA. 2016a. Beverages sales in Mexico before and after implementation of a sugar sweetened beverage tax. PLoS One 11: e0163463. 27668875 10.1371/journal.pone.0163463 PMC5036812

[czaa168-B13] Colchero MA , Popkin BM, Rivera JA, Ng SW. 2016b. Beverage purchases from stores in Mexico under the excise tax on sugar sweetened beverages: observational study. British Medical Journal **352**: h6704. 26738745 10.1136/bmj.h6704 PMC4986313

[czaa168-B14] Colchero MA , Rivera-Dommarco J, Popkin BM, Ng SW. 2017. In Mexico, evidence of sustained consumer response two years after implementing a sugar-sweetened beverage tax. Health Affairs 36: 564–71. 28228484 10.1377/hlthaff.2016.1231 PMC5442881

[czaa168-B15] Cominato L , Di Biagio GF, Lellis D et al 2018. Obesity prevention: strategies and challenges in Latin America. Current Obesity Reports 7: 97–104. 29737493 10.1007/s13679-018-0311-1

[czaa168-B16] Curti D , Shang C, Ridgeway W, Chaloupka FJ, Fong GT. 2015. The use of legal, illegal and roll-your-own cigarettes to increasing tobacco excise taxes and comprehensive tobacco control policies: findings from the ITC Uruguay Survey. Tobacco Control 24: iii17–24. 25740084 10.1136/tobaccocontrol-2014-051890 PMC4605142

[czaa168-B17] Delipalla S , O'Donnell O. 1998. *The Comparison Between Ad Valorem and Specific Taxation under Imperfect Competition: Evidence from the European Cigarette Industry*. Department of Economics Discussion Paper, No. 9802, University of Kent, Department of Economics, Canterbury.

[czaa168-B18] Duffey K , Gordon-Larsen P, Shikany J et al 2010. Food price and diet and health outcomes: 20 years of the CARDIA study. Archives of Internal Medicine 170: 420–6. 20212177 10.1001/archinternmed.2009.545 PMC3154748

[czaa168-B19] Encuesta Mensual de la Industria Manufacturera (EMIM). 2019. https://www.inegi.org.mx/app/buscador/default.html? q=EMIM, accessed August 2018.

[czaa168-B20] Ferrante D , Levy D, Peruga A, Compton C, Romano E. 2007. The role of public policies in reducing smoking prevalence and deaths: the Argentina Tobacco Policy Simulation Model. Revista Panamericana de Salud Pública 21: 37–49. 17439692 10.1590/s1020-49892007000100005

[czaa168-B21] Garcés A , Garcés M, Barnoya J et al 2014. Conference report on tobacco taxes in Central America: current situation and opportunities to reduce prevalence and increase fiscal revenues. Nicotine & Tobacco Research 16: S65–70. 24343958 10.1093/ntr/ntt048

[czaa168-B22] Gigliotti A , Figueiredo VC, Madruga CS et al 2014. How smokers may react to cigarette taxes and price increases in Brazil: data from a national survey. BMC Public Health 14:327. 24712903 10.1186/1471-2458-14-327 PMC3991916

[czaa168-B23] Gonzalez-Rozada M , Ramos-Carbajales A. 2016. Implications of raising cigarette excise taxes in Peru. Revista Panamericana de Salud Publica **40:** 250–5. 28001201

[czaa168-B24] Goodchild M , Perucic A, Nargis N. 2016. Modelling the impact of raising tobacco taxes on public health and finance. Bulletin of the World Health Organization 94: 250–7. 27034518 10.2471/BLT.15.164707 PMC4794304

[czaa168-B25] Goodchild M , Sandoval R, Belausteguigoitia I. 2017. Generating revenue by raising tobacco taxes in Latin America and the Caribbean. Revista Panamericana de Salud Pública **41**: e151. 10.26633/RPSP.2017.151 PMC6645199 31384270

[czaa168-B26] Guerrero-Lopez C , Munos-Hernandez J, de Miera-Juarez B, Reynales-Shigematsu L. 2013. Tobacco consumption, mortality and fiscal policy in Mexico. Salud Publica de Mexico 55: 2. 24626704

[czaa168-B27] Heller PS. 2006. The prospects of creating “fiscal space” for the health sector. Health Policy and Planning 21: 75–9. 16415338 10.1093/heapol/czj013

[czaa168-B28] Hernández F M , Batis C, Rivera JA, Arantxa Colchero M. 2019. Reduction in purchases of energy-dense nutrient-poor foods in Mexico associated with the introduction of a tax in 2014. Preventive Medicine 118: 16–22. 30287330 10.1016/j.ypmed.2018.09.019 PMC6322966

[czaa168-B29] Higgins JPT , Thomas J, Chandler J et al 2019. Cochrane Handbook for Systematic Reviews of Interventions version 6.0 (updated July 2019). *Cochrane*. www.training.cochrane.org/handbook, accessed August 2018.

[czaa168-B30] Iglesias R. 2016. Increasing excise taxes in the presence of an illegal cigarette market: the 2011 Brazil tobacco reform. Revista Panamericana de Salud Publica 40: 243–9. 28001200

[czaa168-B31] Iglesias R , Szklo A, Souza M, de Almeida L. 2017. Estimating the size of illicit tobacco consumption in Brazil: findings from the global adult tobacco survey. Tobacco Control 26: 53–9. 26797750 10.1136/tobaccocontrol-2015-052465

[czaa168-B32] James EK , Saxena A, Franco Restrepo C et al 2019. Distributional health and financial benefits of increased tobacco taxes in Colombia: results from a modelling study. Tobacco Control 28: 374–80. 30093415 10.1136/tobaccocontrol-2018-054378

[czaa168-B33] Jan C , Lee M, Roa R et al 2014. The association of tobacco control policies and the risk of acute myocardial infarction using hospital admissions data. PLoS One 9: e88784. 24520421 10.1371/journal.pone.0088784 PMC3919809

[czaa168-B34] Jha P , Chaloupka FJ. 2000. The economics of global tobacco control. BMJ 321: 358–61. 10926598 10.1136/bmj.321.7257.358 PMC1118333

[czaa168-B35] Jimenez-Ruiz J , de Miera B, Reynales-Shigematsu L, Waters H, Hernandez-Avila M. 2008. The impact of taxation on tobacco consumption in Mexico. Tobacco Control 17: 105–10. 18285383 10.1136/tc.2007.021030

[czaa168-B4806048] Kain J , Concha F, Moreno L, Leyton B. 2014. School-based obesity prevention intervention in chilean children: effective in controlling, but not reducing obesity. Journal of Obesity 2014: 1–8. 10.1155/2014/618293 PMC4020193 24872892

[czaa168-B36] Kostova D , Chaloupka FJ, Yurekli A, on behalf of the GATS Collaborative Group et al 2014. A cross-country study of cigarette prices and affordability: evidence from the Global Adult Tobacco Survey. Tobacco Control 23: e3. 10.1136/tobaccocontrol-2011-050413 22923477

[czaa168-B37] Levy DT , Tam J, Kuo C, Fong GT, Chaloupka F. 2018. The impact of implementing tobacco control policies. Journal of Public Health Management and Practice 1: 1. 10.1097/PHH.0000000000000780 PMC6050159 29346189

[czaa168-B38] Maldonado N , Llorente B, Deaza J. 2016. Impuestos y demanda de cigarrillos en Colombia. Rev Panam Salud Publica 40: 229–36. 28001198

[czaa168-B39] Malik VS , Pan A, Willett WC, Hu FB. 2013. Sugar-sweetened beverages and weight gain in children and adults: a systematic review and meta-analysis. The American Journal of Clinical Nutrition 98: 1084–102. 23966427 10.3945/ajcn.113.058362 PMC3778861

[czaa168-B40] Martinez E , Mejia R, Pérez-Stable E. 2015. An empirical analysis of cigarette demand in Argentina. Tobacco Control 24: 89–93. 23760657 10.1136/tobaccocontrol-2012-050711 PMC4102660

[czaa168-B41] Mejia R , Schoj V, Barnoya J, Flores ML, Pérez-Stable EJ. 2008. Tobacco industry strategies to obstruct the FCTC in Argentina. CVD Prevention and Control 3: 173–9. 19956349 10.1016/j.cvdpc.2008.09.002 PMC2630219

[czaa168-B0529512] Muller F. 2008. Smoking and smoking cessation in Latin America: a review of the current situation and available treatments. International Journal of Chronic Obstructive Pulmonary Disease 3: 285–93. 18686737 10.2147/copd.s2654 PMC2629971

[czaa168-B42] Nakamura R , Mirelman AJ, Cuadrado C et al 2018. Evaluating the 2014 sugar-sweetened beverage tax in Chile: an observational study in urban areas. PLOS Medicine 15: e1002596. 29969456 10.1371/journal.pmed.1002596 PMC6029775

[czaa168-B43] Nielsen’s Mexico Consumer Panel Services. 2019. https://www.nielsen.com/ssa/en/solutions/capabilities/consumer-panels/, accessed August 2018.

[czaa168-B44] Ng SW , Rivera JA, Popkin BM, Colchero MA. 2019. Did high sugar-sweetened beverage purchasers respond differently to the excise tax on sugar-sweetened beverages in Mexico? Public Health Nutrition 22: 750–7. 10.1017/S136898001800321X PMC6581622 30560754

[czaa168-B45] Ortega-Avila A , Papadaki A, Jago R. 2018. Exploring perceptions of the Mexican sugar-sweetened beverage tax among adolescents in north-west Mexico: a qualitative study. Public Health Nutrition 21: 618–26. 29061203 10.1017/S1368980017002695 PMC10261074

[czaa168-B46] Pan-American Health Organisation (PAHO). 2015. Fiscal space for increasing health priority in public spending in the Americas Region; Washington, DC, Pan American Health Organisation.

[czaa168-B47] Paoletti L , Jardin B, Carpenter MJ, Cummings KM, Silvestri GA. 2012. Current Status of Tobacco Policy and Control. Journal of Thoracic Imaging 27: 213–9. 22847588 10.1097/RTI.0b013e3182518673 PMC3409436

[czaa168-B48] Pindyck R , Rubinfeld DL. 2018. Microeconomics. 9th edn. New York, NY: Pearson.

[czaa168-B49] Redondo M , Hernández-Aguado I, Lumbreras B. 2018. The impact of the tax on sweetened beverages: a systematic review. The American Journal of Clinical Nutrition 108: 548–63. 30535085 10.1093/ajcn/nqy135

[czaa168-B50] Reynales-Shigematsu LM , Fleischer NL, Thrasher JF et al 2015. Effects of tobacco control policies on smoking prevalence and tobacco-attributable deaths in Mexico: the SimSmoke model. Revista Panamericana de Salud Publica 38: 316–25. 26758223

[czaa168-B51] Rodríguez-Iglesias G , Schoj V, Chaloupka F, Champagne B, González-Rozada M. 2017. Analysis of cigarette demand in Argentina: the impact of price changes on consumption and government revenues. Salud Pública de México 59: 95–101. 28423115 10.21149/7861

[czaa168-B52] Rodriguez-Iglesias G , Ríos B, Shammah C, Schoj V. 2016. State of affairs regarding fiscal and affordability aspects of tobacco in Argentina. Revista Argentina de Cardiología 84: 140–4.

[czaa168-B53] Saenz-de-Miera B , Thrasher J, Chaloupka F et al 2010. Self-reported price of cigarettes, consumption and compensatory behaviours in a cohort of Mexican smokers before and after a cigarette tax increase. Tobacco Control 19: 481–7. 20870740 10.1136/tc.2009.032177 PMC2991075

[czaa168-B54] Sánchez-Romero L , Penko J, Coxson P et al 2016. Projected impact of Mexico’s sugar sweetened beverage tax policy on diabetes and cardiovascular disease: a modeling study. PLoS Medicine 13: e1002158. 27802278 10.1371/journal.pmed.1002158 PMC5089730

[czaa168-B55] Sandoval RC , Belausteguigoitia I, Anselm H. 2016. The case of tobacco taxation: where we are and how to accelerate its use for public health. Revista Panamericana de Salud Publica 40: 200–1. 28001193

[czaa168-B57] Sterne JA , Hernán MA, Reeves BC et al 2016. ROBINS-I: a tool for assessing risk of bias in non-randomised studies of interventions. British Medical Journal **355**: i4919. 10.1136/bmj.i4919 PMC5062054 27733354

[czaa168-B58] Szklo A , Iglesias R, Carvalho de Souza M, Szklo M, Maria de Almeida L. 2018. Trends in illicit cigarette use in Brazil estimated from legal sales, 2012–2016. American Journal of Public Health 108: 265–9. 29267067 10.2105/AJPH.2017.304117 PMC5846577

[czaa168-B59] Taillie L , Rivera J, Popkin B, Batis C. 2017. Do high vs. low purchasers respond differently to a nonessential energy-dense food tax? Two-year evaluation of Mexico's 8% nonessential food tax. Preventive Medicine 105: S37–42. 10.1016/j.ypmed.2017.07.009 PMC5732875 28729195

[czaa168-B60] U.S. Department of Health and Human Services. The Health Consequences of Smoking. 2014. 50 Years of Progress. A Report of the Surgeon General. Atlanta, GA: U.S. Department of Health and Human Services, Centers for Disease Control and Prevention, National Center for Chronic Disease Prevention and Health Promotion, Office on Smoking and Health.

[czaa168-B61] White JS , Ross H. 2015. Smokers’ strategic responses to sin taxes: evidence from panel data in Thailand. Health Economics 24: 127–41. 24677731 10.1002/hec.3004 PMC3989462

[czaa168-B62] Whitehead R , Brown L, Riches E et al 2018. *Rapid Evidence Review: Strengths and Limitations of Tobacco Taxation and Pricing Strategies*. NHS Health Scotland, WHO Collaborating Centre for Health Promotion and Public Health Development.

[czaa168-B63] World Bank. 2019. *Current Health Expenditure (% of GDP)—Latin America & Caribbean*. https://data.worldbank.org/indicator/SH.XPD.CHEX.GD.ZS?contextual=default&locations=ZJ.

[czaa168-B64] World Health Organisation. 2003. *Tobacco Framework Convention on Tobacco Control*. https://www.who.int/tobacco/framework/fctc_en.pdf?ua=1.

[czaa168-B65] World Health Organisation. 2004. *The Establishment and Use of Dedicated Taxes for Health*. World Health Organisation, Regional Office for the Western Pacific**:** 80. https://www.who.int/health_financing/documents/dedicated_taxes.pdf?ua=1

[czaa168-B66] World Health Organisation. 2015. *Tobacco Control in Mexico*. https://www.who.int/tobacco/about/partners/bloomberg/mex/en/.

[czaa168-B67] Wright A , Smith KE, Hellowell M. 2017. Policy lessons from health taxes: a systematic review of empirical studies. BMC Public Health 17:583. 28629470 10.1186/s12889-017-4497-z PMC5477308

