# Supplementary material for: Sin taxes and their effect on consumption, revenue generation and health improvement: a systematic literature review in Latin America
Source: Health Policy Plan. 2021 Apr 22;36(5):790–810. doi: 10.1093/heapol/czaa168 (PMC8173601; doi:10.1093/heapol/czaa168)
Supplement: czaa168_Supp [file czaa168_supp.zip › Appendix Table 1_Risk_of_Bias_Assessment_Details_FINAL_27AUG2020.docx]

**Appendix Table 1 – Sin taxes in the Latin American context: Details of risk of bias assessment**

| **No.** | **Study name** | **Confounding Bias^1^** | **Selection of participants bias^2^** | **Intervention classification bias^3^** | **Deviation from intended intervention bias^4^** | **Missing data bias^5^** | **Outcome measurement bias^6^** | **Selection of reported result bias^7^** |
| --- | --- | --- | --- | --- | --- | --- | --- | --- |
| 1 | Alvarez-Sanchez et al. (2018) | Low risk | Low risk | Low risk | High risk: **2** | Low risk | High risk: **1** | Low risk |
| 2 | Bardach et al. (2016) | Low risk | Low risk | Low risk | Low risk | High risk: **2** | High risk: **2** | Low risk |
| 3 | Batis et al. (2016) | Low risk | Low risk | Low risk | High risk: **1** | High risk: **2** | Low risk | Low risk |
| 4 | Caro et al. (2018) | Low risk | Low risk | Low risk | Low risk | High risk: **2** | Low risk | Low risk |
| 5 | Chàvez (2016) | Low risk | Medium risk:**1,2** | Medium risk: **2** | High risk: **1; 2** | Low risk | Low risk | Low risk |
| 6 | Claro, Popkin et al. (2012) | Low risk | Low risk | Low risk | Low risk | Low risk | Low risk | Low risk |
| 7 | Colchero, Guerrero et al. (2016) | Low risk | Low risk | Medium risk: **1** | High risk: **1** | Medium risk: **2** | High risk: **2** | Low risk |
| 8 | Colchero, Popkin et al. (2016) | Low risk | Low risk | Low risk | Low risk | High risk: **2** | High risk: **2** | Low risk |
| 9 | Colchero, Rivera et al. (2017) | High risk: **1** | High risk: **1** | Low risk | Medium risk:  **1** | High risk: **2** | High risk: **2** | Low risk |
| 10 | Curti et al. (2005) | Low risk | Low risk | Medium risk: **1** | High risk:**1; 2** | Low risk | Medium risk: **1** | Medium risk: **2** |
| 11 | Ferrante et al. (2007) | Low risk | Medium risk: **1** | Low risk | Low risk | Low risk | Medium risk: **2** | Low risk |
| 12 | Garces et al. (2014) | N/A | N/A | N/A | Low risk | Low risk | Low risk | Low risk |
| 13 | Gonzalez-Rozada et al. (2016) | Low risk | Low risk | Low risk | Low risk | Low risk | Medium risk: **2** | Low risk |
| 14 | Goodchild et al. (2017) | Low risk | Low risk | Low risk | Low risk | Low risk | Medium risk: **2** | Low risk |
| 15 | Guerrero-Lopez et al. (2013) | Low risk | Low risk | Low risk | Low risk | Low risk | High risk: **1** | Low risk |
| 16 | Hernandez et al. (2019) | Low risk | Low risk | Low risk | High risk: **2** | Low risk | Low risk | Low risk |
| 17 | Iglesias (2016) | N/A | N/A | N/A | Low risk | Low risk | Low risk | Medium risk: **1** |
| 18 | Iglesias, Skzlo et al. (2016) | Low risk | Low risk | Low risk | Low risk | Medium/unclear:**2** | High risk: **1** | High risk: **2** |
| 19 | Jan et al. (2014) | Low risk | Low risk | Low risk | Low risk | High risk: **2** | Low risk | Low risk |
| 20 | Jiménez- Ruiz et al. (2008) | Low risk | Low risk | Low risk | Medium risk: **1** | Low risk | Medium risk: **2** | Low risk |
| 21 | James et al. (2019) | Low risk | Low risk: | Low risk | Low risk | Low risk | Low risk | Low risk |
| 22 | Kostova et al. (2014) | Low risk | Low risk | Low risk | Low risk | Low risk | High risk: **1** | Low risk |
| 23 | Maldonado et al. (2016) | Low risk | Low risk | Low risk | Low risk | Low risk | Low risk | Low risk |
| 24 | Martinez et al. (2013) | N/A | N/A | N/A | Low risk | Low risk | Low risk | Low risk |
| 25 | Nakamura et al. (2018) | Low risk | Low risk: | Low risk | High risk: **1** | Low risk | Low risk | Low risk |
| 26 | Ng et al. (2018) | Medium risk | Low risk | Low risk | High risk: **1,2** | Low risk | High risk: **1** | High risk: **2** |
| 27 | Ortega et al. (2017) | Low risk | High risk: **1** | Low risk | Low risk | Low risk | Low risk | Low risk |
| 28 | Reynales-Shige-matsu et al. (2015) | Low risk | Low risk | Low risk | Low risk | Low risk | Medium risk: **2** | Low risk |
| 29 | Rodriguez-Igle-sias, Rios et al. (2016) | N/A | N/A | N/A | Low risk | Low risk | Low risk | Low risk |
| 30 | Rodriguez-Igle-sias, Schoj et al. (2017) | Low risk | Low risk | Low risk | Low risk | Low risk | High risk: **2** | Low risk |
| 31 | Saenz-de-Miera, et al. (2010) | Low risk | Medium risk:**3;1** | Low risk | High risk: **1;2** | High risk: **2** | Low risk | Low risk |
| 32 | Sanchez- Romero et al. (2016) | Low risk | Low risk | Low risk | Low risk | High risk: **2** | High risk: **2** | Low risk |
| 33 | Szklo et al. (2018) | Low risk | Low risk | Low risk | Low risk | High risk: **1** | High risk: **1** | Low risk |
| 34 | Taillie et al. (2017) | Low risk | Low risk | Low risk | Low risk | High risk: **2** | Low risk | Low risk |

Notes: ^1^ **Confounding bias:** Baseline confounding occurs when one or more prognostic variables (factors that predict the outcome of interest) also predicts the interventions received at baseline. ROBINS-I can also address time-varying confounding, which occurs when individuals switch between the interventions being compared and when post-baseline prognostic factors affect the intervention received after baseline**.** High risk of bias in this domain is related to unclear definition and distinction of variables; can be due to: **1: confounding caused by concurring taxes implemented.**

^2^ **Selection of participant bias:** When exclusion of some eligible participants, or the initial follow-up time of some participants, or some outcome events is related to both intervention and outcome, there will be an association between interventions and outcome even if the effects of the interventions are identical. This form of selection bias is distinct from confounding – A specific example is bias due to the inclusion of prevalent users, rather than new users, of an intervention. High risk of bias in this domain is related to unclear definition of the sample, interventions, outcomes and follow-up procedures; can be sub-categorised in: **1: limited sample of participants; 2: too short or no follow-up; 3: No blinding of participants.**

^3^ **Intervention classification bias:** Bias introduced by either differential or non-differential misclassification of intervention status. Non-differential misclassification is unrelated to the outcome and will usually bias the estimated effect of intervention towards the null. Differential misclassification occurs when misclassification of intervention status is related to the outcome or the risk of the outcome and is likely to lead to bias**.** High risk of bias in this domain is related to wrong estimation of intervention; can be due to: **1: Misclassification of intervention categories; 2: Timing misclassifications.**

^4^ **Deviation from intended intervention bias:** Bias that arises when there are systematic differences between experimental intervention and comparator groups in the care provided, which represent a deviation from the intended intervention(s). Assessment of bias in this domain will depend on the type of effect of interest (either the effect of assignment to intervention or the effect of starting and adhering to intervention). High risk of bias in this domain is related to relevant deviation between intervention and comparator groups; can be due to: **1: Incomplete or wrong comparators definitions; 2: No control groups.**

^5^ **Missing data bias:** Bias that arises when latter follow-up is missing for individuals initially included and followed (such as differential loss to follow-up that is affected by prognostic factors); bias due to exclusion of individuals with missing information about intervention status or other variables such as confounders. High risk of bias in this domain is related to poor or weak data collection design; can be due to**: 1: self-reported data; 2: incomplete or no data available.**

^6^ **Outcome measurement bias:** Bias introduced by either differential or non-differential errors in measurement of outcome data. Such bias can arise when outcome assessors are aware of intervention status, if different methods are used to assess outcomes in different intervention groups, or if measurement errors are related to intervention status or effects. High risk of bias in this domain is related to errors in assessing outcomes due to detection bias or to the study design, this can be sub-categorised in: **1: under or overestimation due to self-reporting; 2: Incomplete or vague assessment.**

^7^ **Selection of reported results bias**: Selective reporting of results in a way that depends on the findings and prevents the estimate from being included in a meta-analysis (or other synthesis). High risk of bias in this domain is related to selective reporting leading to bias; this can be sub-categorised in: **1: Non-replicable results; 2: Exclusion of some results.**

N/A: not applicable.

Source: The authors based on the literature.
